# Discriminative graph regularized representation learning for recognition

**DOI:** 10.1371/journal.pone.0326950

**Published:** 2025-07-17

**Authors:** Jinshan Qi, Rui Xu

**Affiliations:** 1 School of Computer Science and Technology, Huaiyin Normal University, Huaian, China; 2 School of Information, Renmin University of China, Beijing, China; Universidad de Granada, SPAIN

## Abstract

Feature extraction has been extensively studied in the machine learning field as it plays a critical role in the success of various practical applications. To uncover compact low-dimensional feature representations with strong generalization and discrimination capabilities for recognition tasks, in this paper, we present a novel discriminative graph regularized representation learning (DGRL) model that is able to elegantly incorporate both global and local geometric structures as well as the label structure of data into a joint framework. Specifically, DGRL first integrates dimension reduction into ridge regression rather than treated them as two irrelevant steps, which enables us to capture the underlying subspace structure and correlation patterns among classes. Additionally, a graph regularizer that fully utilizes the local class information is developed and introduced to the new framework so as to enhance the classification accuracy and prevent overfitting. A kernel version of DGRL, called KDGRL, is also established for dealing with complex nonlinear data by using the kernel trick. The proposed framework naturally unifies several well-known approaches and elucidates their intrinsic relationships. We provide detailed theoretical derivations of the resulting optimization problems of DGRL and KDGRL. Meanwhile, we design two simple and tractable parameter estimation procedures based on cross-validation technique to speed up the model selection processes for DGRL and KDGRL. Finally, we conduct comprehensive experiments on diverse benchmark databases drawn from different areas to evaluate the proposed theories and algorithms. The results well demonstrate the effectiveness and superiority of our methods.

## Introduction

Learning a compact and powerful discriminative representation of high-dimensional data has shown remarkable importance to perform accurate and efficient regression or classification because of the so-called curse of dimensionality [1]. Over the past decades, subspace learning based dimension reduction has been extensively studied and widely used in extracting discriminative feature representations by removing redundant, irrelevant, and noisy information. Two of the most well-known techniques on this topic are principal component analysis (PCA) [1] and linear discriminant analysis (LDA) [2].

LDA pursues a linear discriminative subspace with minimum within-class dispersion and maximum between-class separation. Classical LDA always suffers from the small sample size (SSS) problem, which is known to cause serious stability problems for LDA. Consequently, many research works [3–5] have been developed to address this issue in recent decades. Moreover, since the learned projective functions of PCA and LDA are linear combinations of all the original features, they may fail to discover essential data structure that is nonlinear. Kernel based technique [6] provides powerful and tractable extensions of linear models to nonlinear cases, and it has been successfully used to extend PCA and LDA to the kernel-induced feature space [7–9]

Traditional PCA and LDA only consider the global structure of data, while the local structure, which is often important for many real applications [10–12], is ignored. To this end, a variety of manifold-based techniques [13–16] has been developed to uncover the geometrical structure of the underlying manifold. The core idea of these methods is to construct the affinity graph via different measure metrics and then maintain the desired local neighborhood relations characterized by the graph in a new space. The typical application of manifold learning is the graph embedding which has been widely used to address the issue of overfitting for recognition tasks by enhancing the intra-class compactness recently [17–20].

Many of the popular subspace learning techniques such as LDA, locality preserving projection (LPP), and their kernel extensions can also be formulated as a least squares regression (LSR) problem [2]. The LSR-type formulation is not only readily and flexible to introduce various regularization techniques to improve the interpretation and generalization ability of the resulting model, but also provides efficient and scalable implementations by many existing iterative algorithms. Most of the above methods are commonly applied as a separate data preprocessing procedure. As a consequence, feature learning and classification are often treated as two irrelevant steps, and hence, the overall optimum of the algorithms cannot be guaranteed. Motivated by the recent development of low-rank representation (LRR) [21], low-rank constraint has been incorporated into LSR [22–24], which can globally preserve the membership of data and capture the underlying correlations behind samples. Fang et al. [25] introduced a robust latent subspace learning (RLSL) method by simultaneously minimizing the regression loss and reconstruction error so as to enhance the connection between the learned data representation and the classification performance. Supervised approximate low-rank projection learning (SALPL) [26] shares the similar idea of RLSL, in which projection learning process of latent low-rank representation is integrated into rigid regression. Although these methods are very effective in learning an informative feature representation, they only exploit the global structure of data while ignoring the local structure.

Recent studies [10,12] show that local and global structures are indispensable and complement to each other in learning discriminative representation for image classification. In view of this, various subspace learning techniques integrating the theory of graph embedding [27], sparse representation (SR) [28], or LRR [18] have been developed to expose the inherent structure of data. For example, low-rank sparse preserving projections (LSPP) [27] combines manifold learning with low-rank sparse representation so that the self-expressiveness property and the intrinsic geometric structure of data points in the embedding space can be better revealed. It is noted that low-rank criterion governs the global relation of the entire data set, whereas sparsity criterion dominates the local structure around each individual data point [10].

The bring forward of the above methods greatly promote the development of feature learning theory and practice. Nonetheless, they face some challenges that have not been exhaustively explored and perfectly solved as well. First, since these methods are modified with different motivations by introducing extra regularization terms or constraints, the corresponding objective functions usually become non-convex and even non-smooth. Such optimization problems are generally solved with a quite complicated alternating procedure, e.g., employing the augmented Lagrange multiplier (ALM) algorithms [29], which inevitably involve iterative processes and easily converge to local minimums. What is more, theoretically, the convergence of the ALM style optimization approach cannot be well guaranteed in the cases of more than two blocks of primary variables. Thus, most of these methods have to validate their convergence properties via experiments. On the other hand, the choice of regularization parameters, which is key to the success of these methods, has been traditionally entirely left to the user. In practice, cross validation is a widely used evaluation technique for model selection to obtain a satisfactory performance. Unfortunately, it is often computationally prohibitive to make an optimal tuning for the many regularization parameters from a large set of candidates, especially in high-dimensional data analysis. Finally, although these methods work well for linear problems, they may be less effective when severe nonlinearity is involved.

In light of these deficiencies, a novel method termed as DGRL is proposed in this work to learn an appropriate data representation by discovering a latent subspace for multicategory classification. DGRL smoothly integrates feature extraction and recognition into a joint optimization framework rather than performing them in two independent steps, which guarantees an overall optimum. Moreover, DGRL directly introduces the underlying discriminative and geometrical information into a general graph regularization to guide the projection learning and the training of classifier, which encourages the method to obtain a better performance. The kernelized counterpart of DGRL (KDGRL) is also developed to boost the nonlinear representation of samples. We make an insightful and systematic investigation of the problem of regularization parameter estimation in both DGRL and its kernel version.

Our key contributions are summarized as follows.

1) We combine feature learning and classification with some constraints to form a unified framework. Under this framework, we present two models, i.e., DGRL and KDGRL, in which both the global Euclidean and local manifold structures of data as well as the underlying class information are exploited simultaneously and naturally evolved together to learn a more compact and discriminative subspace for dimension reduction and classification.
2) We design a supervised graph regularizer that elegantly incorporates several informative constraints, such as local consistency and discriminative property, to enforce the uncovered data representation in the transformed category space more competent to the subsequent classification.
3) We show that the matrix computations involved in DGRL and KDGRL can be simplified, and we develop two simple yet effective model selection algorithms for them which can greatly accelerate the cross validation process, especially for high-dimensional, small sample size data.

The remainder of this paper is organized as follows. In the Methods section, we first present the motivation and derivation of DGRL in detail, and then provide the model selection algorithm for DGRL with algorithmic description. We then extend DGRL to a kernel version for nonlinear feature extraction and recognition. Extensive experiments are conducted and analyzed In the experimental section. Conclusion section draws conclusions and discusses future works.

## Proposed methods

### Notations

Throughout the paper, scalars, vectors, and matrices are written as italic letters, bold lowercase letters, and bold uppercase letters, respectively. Let $𝐃\in {\mathbb{R}}^{d\times n}$ be the original data matrix consisting of *n* samples in the *d*-dimensional space. Let $𝐆\in {\mathbb{R}}^{n\times k}$represent the binary class label matrix corresponding to **D**, where *k* is the number of classes, and k≥2. Specifically, if the *i*th sample is from the *j*th class, *g*_*i j*_ = 1; otherwise, *g*_*i j*_ = 0, where *g*_*i j*_ denotes the (*i*, *j*)th element of **G**. To be clear, we first define $𝐗=𝐃({𝐈}_{n}-\frac{1}{n}1_{n}1_{n}^{T}in{\mathbb{R}}^{d\times n}$ as the centered data matrix such that the global mean is zero, i.e., $\sum_{i=1}^{n}{𝐱}_{i}=0$, where ${𝐈}_{n}$ is an identity matrix of size *n*, $1_{n}\in {\mathbb{R}}^{n}$ is a vector of all ones, and ${𝐱}_{i}\in {\mathbb{R}}^{d}$ represents the *i*th column of **X**. We also define the normalized class label matrix as $𝐘=𝐆{\left({𝐆}^{T}𝐆\right)}^{-\frac{1}{2}}\in {\mathbb{R}}^{n\times k}$ with $yij=1/\sqrt{n_{j}}$ while ${𝐱}_{i}$ belongs to the *j*th class and *y*_*ij*_ = 0 otherwise, where *n*_*j*_ is the sample size of the *j*th class, $\sum_{j=1}^{k}n_{j}=n$, and n≫k.

### Motivation and formulation

We present a regularized joint learning framework for simultaneous feature extraction and multicategory classification in a supervised manner, which integrates both global and local structures to uncover the most compact and discriminative subspace so as to facilitate the follow-up recognition tasks. In the proposed framework, the original high-dimensional feature space and its underlying semantic space are linked by a linear transformation $𝐐\in {\mathbb{R}}^{d\times s}$ that projects each datapoint ${𝐱}_{i}$, for 1≤i≤n, in the *d*-dimensional space onto a *s*-dimensional space as ${𝐐}^{T}{𝐱}_{i}$, where, typically, s≪d. To improve the overall generalization and discriminative abilities of the algorithm, we aim to employ the extracted features ${𝐐}^{T}𝐗$ as the new discriminate representations to build a bridge between the original features and objective outputs so that they are seamlessly connected. In addition, the high-level semantic label information together with the geometric property of data are incorporated into our framework in the form of graph based regularization constraint by virtue of manifold learning. This formulates the following optimization problem


$$\underset{𝐖,𝐐}{\min}\sum_{j=1}^{k}\sum_{i=1}^{n}ℒ\left(y_{ij},f_{j}({𝐱}_{i})\right)+\lambda \mathrm{\backslash Upomega}\left(f\right)+\alpha \mathrm{\backslash Uppsi}\left(f\right)$$ (1)

where the loss function ℒ measures the approximate error between the desired and actual outputs, the regularization term \Upomegacontrols the model complexity, and the supervised graph regularization term \Uppsi is specially designed for classification purpose with a strong discrimination capability, which effectively encodes the locality and similarity among data points by making the best of class information so that the separability of samples from different classes in the category space can be further enhanced. Additionally, λ≥0 and α≥0 are two nonnegative regularization parameters for balancing respective terms, and $f_{j}({𝐱}_{i})$ indicates that the extracted discriminative features ${𝐐}^{T}𝐗$ are fed into the specified classifier $f_{j}$ to build the mapping relation **W** between the latent subspace **Q** and the label space **Y**.

By combining appropriate regularization or relaxed labels [30–32], the least squares loss function has been shown to be comparable to other loss functions such as the logistic loss and hinge loss. In this work, we simply adopt least squares loss $\begin{array}{c} ℒ\left(y_{ij},f_{j}({𝐱}_{i})\right)=(f_{j}\left({𝐱}_{i}\right)-y_{ij}{)}^{2} \end{array}$ as well as $\ell_{2}$-norm regularization $\mathrm{\backslash Upomega}\left(f\right)=\sum_{j=1}^{k}{\left|\left|{𝐰}_{j}\right|\right|}_{2}^{2}$ to learn a set of *k* linear models, $f_{j}:𝐱\in {ℜ}^{d}\to f_{j}\left(𝐱\right)={𝐰}_{j}^{T}{𝐐}^{T}𝐱$, j=1,⋯,k, by minimizing the classification error. In this way, the embedding representation will be tightly coupled with classification. Accordingly, the objective function (1) becomes


$$\min_{𝐖,𝐐}{\left|\left|{𝐗}^{T}QW-𝐘\right|\right|}_{F}^{2}+\lambda {\left|\left|𝐖\right|\right|}_{F}^{2}+\alpha \mathrm{\backslash Uppsi}\left(f\right)$$ (2)

where **W** =$[{𝐰}_{1},{𝐰}_{2},\cdots ,{𝐰}_{k}]\in {\mathbb{R}}^{s\times k}$ and 𝐐 are to be estimated, ${𝐰}_{j}\in {\mathbb{R}}^{s}$ is the weight vector, and ${\left|\left|\cdot \right|\right|}_{F}$ refers to the Frobenius norm of matrix.

Generally, the $\ell_{2}$-norm constraint on ${𝐰}_{j}$ merely emphasizes the smoothness of the function which may not be sufficient for discrimination among classes, since it has little to do with the label information. And more importantly, similar inputs near the decision boundaries are more likely to come from different classes, meaning that the classifier may not be always smooth everywhere, especially for real data sets with complex distributions. Intuitively, a relative ideal classifier should be able to map the data into the target category space where points within each class are close to each other while those belonging to different classes are kept away from each other. Hence, both the underlying discriminative information and the intrinsic geometric structure of the samples are crucial to classification problems. The graph-based supervised dimensionality reduction technique [13–16] provides a practical and feasible approach to fulfil such desired properties, which has already been applied to develop various kinds of algorithms [17,20]. Based on these observations, we explicitly leverage the class information in constructing the graph regularization \Uppsi(f) to simultaneously guide the learning process of the latent subspace **Q** and the linear classifier **W**, thereby endowing them stronger discriminant power. The geometric structure consistency over target semantics and features can be achieved by minimizing the following objective


$$\mathrm{\backslash Uppsi}\left(f\right)=\sum_{i,j=1}^{n}{\left|\left|{𝐖}^{T}{𝐐}^{T}{𝐱}_{i}-{𝐖}^{T}{𝐐}^{T}{𝐱}_{j}\right|\right|}_{2}^{2}S_{ij}$$ (3)

where ${𝐖}^{T}{𝐐}^{T}𝐱$ is the predicted label vector of **x** under the learned projection **QW**, and the discriminating similarity matrix $𝐒=(S_{ij}in{\mathbb{R}}^{n\times n}$ models the local structure of the data manifold as defined in SOLPP [16], that is


$$\begin{array}{c} S_{ij}=\left\{ \begin{array}{c} p{\left(y_{i}\right)}^{2}\exp\left(-\frac{{\left|\left|{𝐱}_{i}-{𝐱}_{j}\right|\right|}_{2}^{2}}{\beta }\right)\left(1+\exp\left(-\frac{{\left|\left|{𝐱}_{i}-{𝐱}_{j}\right|\right|}_{2}^{2}}{\beta }\right)\right)\text{if }{𝐱}_{i}\in N_{K}\left({𝐱}_{j}\right)or{𝐱}_{j}\in N_{K}\left({𝐱}_{i}\right),andy_{i}=y_{j}\\ p\left(y_{i}\right)p\left(y_{j}\right)\exp\left(-\frac{{\left|\left|{𝐱}_{i}-{𝐱}_{j}\right|\right|}_{2}^{2}}{\beta }\right)\left(1-\exp\left(-\frac{{\left|\left|{𝐱}_{i}-{𝐱}_{j}\right|\right|}_{2}^{2}}{\beta }\right)\right)\text{ if }{𝐱}_{i}\in N_{K}\left({𝐱}_{j}\right)or{𝐱}_{j}\in N_{K}\left({𝐱}_{i}\right),andy_{i}\neq y_{j}\\ \;\;0\text{ otherwise } \end{array}\right. \end{array}$$ (4)

where $y_{i}\in \{1,2,\cdots ,k$} is the target label with respect to sample ${𝐱}_{i}$, $p\left(y_{i}\right)=n_{y_{i}}/n$ denotes the prior probability of class $y_{i}$, $N_{K}\left(𝐱\right)$ refers to the set of *K* nearest neighbors of 𝐱, and β is a positive parameter scaling the Euclidean distance between ${𝐱}_{i}$ and ${𝐱}_{j}$. Furthermore, $\exp\left(-{\left|\left|{𝐱}_{i}-{𝐱}_{j}\right|\right|}_{2}^{2}/\beta \right)$, $\begin{array}{c} \left(1+\exp\left(-{\left|\left|{𝐱}_{i}-{𝐱}_{j}\right|\right|}_{2}^{2}/\beta \right)\right) \end{array}$, and $\begin{array}{c} \left(1-\exp\left(-{\left|\left|{𝐱}_{i}-{𝐱}_{j}\right|\right|}_{2}^{2}/\beta \right)\right) \end{array}$ stand for the local weight, the intra-class compactness weight, and the inter-class separability weight, respectively.

According to the celebrated manifold assumption [11], from (3) one can see that the original data geometric structure characterized by **S** is intended to be reflected in the transformed label space. Specifically, minimizing (3) allows to not only separate the nearby data points with the different labels far from each other in the corresponding target space but also constrain that the nearby data points sharing the same label can be kept close to each other after being projected. In this manner, the margins between the samples of different classes at each local neighborhood will be greatly enlarged, and meanwhile the intra-class compactness of the outputs will be intensely boosted, which is beneficial to classification. Besides, the use of **S** equips the graph regularizer \Uppsi(f) with some additional appealing properties like good robustness, margin augmentation and noise suppression. Please see [16] for more details. Further, we can rewrite (3) as


$$\mathrm{\backslash Uppsi}\left(f\right)=tr\left({𝐖}^{T}{𝐐}^{T}XL{𝐗}^{T}QW\right)$$ (5)

where $𝐋\in {\mathbb{R}}^{n\times n}$ is the graph Laplacian matrix and defined as 𝐋=𝐂−𝐒, 𝐂 is a diagonal degree matrix with the *i*th diagonal entry $C_{ii}=\sum_{j}S_{ij}$, and tr($\begin{array}{c} • \end{array}$) is the trace of a square matrix. Note that 𝐋 is symmetric and positive semidefinite.

By incorporating (5) into (2), we obtain the final objective function of our DGRL model as follows:


$$\begin{array}{c} \begin{array}{c} \min\\ 𝐖,𝐐 \end{array}\;\;\;\;{\left|\left|{𝐗}^{T}QW-𝐘\right|\right|}_{F}^{2}+\lambda {\left|\left|𝐖\right|\right|}_{F}^{2}+\alpha tr\left({𝐖}^{T}{𝐐}^{T}XL{𝐗}^{T}QW\right) \end{array}$$ (6)

$$s.t.{𝐐}^{T}𝐐={𝐈}_{s}.$$

Here the orthogonal constraint ${𝐐}^{T}𝐐={𝐈}_{s}$ is imposed to make the problem tractable.

Next, we will show that *s *< min (*n*, *k*) is indeed the dimensionality of the low-rank subspace derived from a generalized discriminant analysis. In this case, the learned mapping **QW** can be regarded as a low-rank projection matrix, since the size of **QW** is d×k, rank(𝐐leqs<d, rank(QWleqs<k. This indicates that the first term in model (6) essentially performs low-rank linear regression, such that the underlying global correlation structures between classes can be well explored [22]. The second term of (6) is to deal with the singularity problem in computing the optimal solution and consequently strengthens the stability of the whole model. The last term of (6) is actually urges the predicted label matrix ${𝐗}^{T}QW$ to reproduce the discriminating similarity structure coded in **L**. During the process, both the within-class compactness and between-class separability of labels in each local area are enhanced, and this helps to alleviate the risk of overfitting [17] while improving the model’s generalization. These factors, when taken together, enable our method to learn a more compact and discriminative subspace **Q** for dimension reduction and classification and naturally differentiate it from previous works [22,24,33,34]. We will establish the corresponding relationships between those algorithms and ours and analyze it in greater detail in the subsequent section.

### The optimal solution

By taking the partial derivative of (6) with respect to **W** and setting it to zero, we have


$$𝐖=({𝐐}^{T}({XX}^{T}+\lambda {𝐈}_{d}+\alpha {XLX}^{T})𝐐{)}^{-1}{𝐐}^{T}XY$$ (7)

Substituting (7) back to (6), it is not difficult to verify that the optimal transformation **Q** can be obtained by solving


$$\begin{array}{c} \min\\ {𝐐}^{T}𝐐={𝐈}_{s} \end{array}tr(({𝐐}^{T}({𝐒}_{t}+\lambda {𝐈}_{d}+\alpha {XLX}^{T})𝐐{)}^{-1}{𝐐}^{T}{𝐒}_{b}𝐐)$$ (8)

where ${𝐒}_{t}={XX}^{T}\in {\mathbb{R}}^{d\times d}$ and ${𝐒}_{b}=𝐗{YY}^{T}{𝐗}^{T}\in {\mathbb{R}}^{d\times d}$ are the total and between-class scatter matrices defined in classical LDA, respectively. One limitation of (8) is that it cannot be directly applied when ${𝐒}_{t}+\lambda {𝐈}_{d}+\alpha {XLX}^{T}$ is singular. To this end, we employ the pseudoinverse in place of the inverse in the optimization in (8), leading to a more general formulation:


$${𝐐}^{*}=\begin{array}{c} \text{argmax}\\ {𝐐}^{T}𝐐={𝐈}_{s} \end{array}tr(({𝐐}^{T}({𝐒}_{t}+\lambda {𝐈}_{d}+\alpha {XLX}^{T})𝐐{)}^{+}{𝐐}^{T}{𝐒}_{b}𝐐)$$ (9)

Note that if the matrix is invertible, its pseudoinverse equals its inverse. A similar criterion has been studied in [4] for a family of generalized LDA algorithms to cope with SSS problem.

To solve (9), we provide the following theorem.

*Theorem 1*: Let **A** be a d×q matrix whose columns are formed by the eigenvectors of $({𝐒}_{t}+\lambda {𝐈}_{d}+\alpha {XLX}^{T}{)}^{+}{𝐒}_{b}$ corresponding to the largest *q* nonzero eigenvalues, where *q* = rank(${𝐒}_{b}$). Let $𝐀=\hat{𝐐}\hat{𝐑}$ be a **QR** factorization of **A**, where $\hat{𝐐}\in {\mathbb{R}}^{d\times q}$ has orthonormal columns and $\hat{𝐑}\in {\mathbb{R}}^{q\times q}$ is upper triangular. Then ${𝐐}^{*}=\hat{𝐐}$ solves the optimization problem (9).

*Proof*: The detailed proof of Theorem 1 is moved to the supplementary file for better flow of the paper.

Theorem 1 implies that the dimension, *s* = *q*, of the subspace transformed by ${𝐐}^{T}$ is at most *k*-1, since the rank of ${𝐒}_{b}$ is bounded from above by *k*-1. Additionally, we can find that the major computation involved in DGRL comes from the eigen-decomposition of $({𝐒}_{t}+\lambda {𝐈}_{d}+\alpha {XLX}^{T}{)}^{+}{𝐒}_{b}$ in solving (9). For high-dimensional data, ${𝐒}_{t}+\lambda {𝐈}_{d}+\alpha {XLX}^{T}$ and ${𝐒}_{b}$ are very large dense matrices of size *d* by *d*, which incur a high computational cost on directly solving such generalized eigenproblem. Moreover, estimating the best regularization parameters λ and αvia cross-validation from a set of candidates will further increase the time complexity sharply. This procedure is often computationally too intensive or even infeasible when the candidate set is large, since it requires expensive matrix computations for each candidate value. We next present an efficient implementation for DGRL, which has the potential to perform model selection from a large search space with low computational effort.

### Model selection for DGRL

Let $𝐗=𝐔\Sigma {𝐕}^{T}$ be the singular value decomposition (SVD) of **X**, where $𝐔\in {\mathbb{R}}^{d\times d}$ and $𝐕\in {\mathbb{R}}^{n\times n}$ are orthogonal, $\Sigma =\text{diag}(\Sigma_{t},0in{\mathbb{R}}^{d\times n}$,$\Sigma_{t}\in {\mathbb{R}}^{t\times t}$ is diagonal and nonsingular with *t* = rank(**X**). Let **U** be partitioned as **U** = (**U**_1_, **U**_2_) such that $={𝐔}_{1}\Sigma_{t}{𝐕}_{1}^{T}$, where **U**_1_$\in {\mathbb{R}}^{d\times t}$, **U**_2_$\in {\mathbb{R}}^{d\times (d-t)}$, and ${𝐕}_{1}\in {\mathbb{R}}^{n\times t}$ contains the first *t* columns of **V**. We start by factoring ${𝐒}_{t}+\alpha {XLX}^{T}$ in the form


$${𝐒}_{t}+\alpha {XLX}^{T}=𝐗\left({𝐈}_{n}+\alpha 𝐋\right){𝐗}^{T}={𝐔}_{1}\Sigma_{t}{𝐕}_{1}^{T}\left({𝐈}_{n}+\alpha 𝐋\right){𝐕}_{1}\Sigma_{t}{𝐔}_{1}^{T}=𝐔\left(\begin{array}{cc} \Sigma_{t}{𝐕}_{1}^{T}\left({𝐈}_{n}+\alpha 𝐋\right){𝐕}_{1}\Sigma_{t} & 0\\ 0 & 0 \end{array}\right){𝐔}^{T}$$ (10)

Denote ${\tilde{\Sigma }}_{t}=\Sigma_{t}{𝐕}_{1}^{T}\left({𝐈}_{n}+\alpha 𝐋\right){𝐕}_{1}\Sigma_{t}\in {\mathbb{R}}^{t\times t}$. It is clear that ${\tilde{\Sigma }}_{t}$ is symmetric and positive definite, for any α≥0. Let ${\tilde{\Sigma }}_{t}=𝐏\Lambda_{t}{𝐏}^{T}$ be the eigen-decomposition of ${\tilde{\Sigma }}_{t}$, where $𝐏\in {\mathbb{R}}^{t\times t}$ is orthogonal and $\Lambda_{t}\in {\mathbb{R}}^{t\times t}$ is diagonal with positive diagonal entries. Then, $({𝐒}_{t}+\lambda {𝐈}_{d}+\alpha {XLX}^{T}{)}^{+}{𝐒}_{b}$ can be expressed as


$$\begin{array}{cc} & ({𝐒}_{t}+\lambda {𝐈}_{d}+\alpha {XLX}^{T}{)}^{+}{𝐒}_{b}={\left(𝐔(\begin{array}{cc} {\tilde{\Sigma }}_{t} & 0\\ 0 & 0 \end{array}){𝐔}^{T}+\lambda 𝐔{𝐈}_{d}{𝐔}^{T}\right)}^{+}{𝐒}_{b}=𝐔{\left(\begin{array}{cc} {\tilde{\Sigma }}_{t}+\lambda {𝐈}_{t} & 0\\ 0 & \lambda {𝐈}_{d-t} \end{array}\right)}^{+}{𝐔}^{T}{𝐒}_{b}\\ =\; & {𝐔}_{1}{\left({\tilde{\Sigma }}_{t}+\lambda {𝐈}_{t}\right)}^{-1}{𝐔}_{1}^{T}{𝐒}_{b}={𝐔}_{1}{\left(𝐏\Lambda_{t}{𝐏}^{T}+\lambda 𝐏{𝐈}_{t}{𝐏}^{T}\right)}^{-1}{𝐔}_{1}^{T}{𝐒}_{b}={𝐔}_{1}𝐏{\left(\Lambda_{t}+\lambda {𝐈}_{t}\right)}^{-1}{𝐏}^{T}{𝐔}_{1}^{T}{𝐒}_{b} \end{array}$$ (11)

where the third equality follows since the null space, **U**_2_, of ${𝐗}^{T}$ also lies in the null space of ${𝐒}_{b}$, i.e., ${𝐔}_{2}^{T}{𝐒}_{b}=0$, and $\Lambda_{t}+\lambda {𝐈}_{t}$ is diagonal and always invertible for any λ≥0.

To simply and efficiently solve the eigen-problem of $\left({𝐒}_{t}+\lambda {𝐈}_{d}+\alpha {XLX}^{T}\right)$, we need the following proposition.

*Proposition 1*: Let a be an eigenvector of $({𝐒}_{t}+\lambda {𝐈}_{d}+\alpha {XLX}^{T}{)}^{+}{𝐒}_{b}$ corresponding to the nonzero eigenvalue μ, then $a={𝐔}_{1}𝐏m$ for some m, where m is an eigenvector of ${\left(\Lambda_{t}+\lambda {𝐈}_{t}\right)}^{-1}{𝐏}^{T}{𝐔}_{1}^{T}{𝐒}_{b}{𝐔}_{1}𝐏$.

*Proof*: Since μ≠0 and a are an eigenvalue-eigenvector pair of $({𝐒}_{t}+\lambda {𝐈}_{d}+\alpha {XLX}^{T}{)}^{+}{𝐒}_{b}$, it follows from (11) that $a=\frac{1}{\mu }{𝐔}_{1}𝐏{\left(\Lambda_{t}+\lambda {𝐈}_{t}\right)}^{-1}{𝐏}^{T}{𝐔}_{1}^{T}{𝐒}_{b}a={𝐔}_{1}𝐏m,$ for some m. We show in the following that m is an eigenvector of ${\left(\Lambda_{t}+\lambda {𝐈}_{t}\right)}^{-1}{𝐏}^{T}{𝐔}_{1}^{T}{𝐒}_{b}{𝐔}_{1}𝐏$. Left-multiplying both sides of ${𝐔}_{1}𝐏{\left(\Lambda_{t}+\lambda {𝐈}_{t}\right)}^{-1}{𝐏}^{T}{𝐔}_{1}^{T}{𝐒}_{b}a=\mu a,$ by ${𝐏}^{T}{𝐔}_{1}^{T}$ yields ${\left(\Lambda_{t}+\lambda {𝐈}_{t}\right)}^{-1}{𝐏}^{T}{𝐔}_{1}^{T}{𝐒}_{b}\left({𝐔}_{1}𝐏m\right)=\mu {𝐏}^{T}{𝐔}_{1}^{T}\left({𝐔}_{1}𝐏m\right)=\mu m.$

This completes the proof of the proposition.

Proposition 1 tells us that with the above calculated $\Lambda_{t}$, ${𝐔}_{1}$, and **P**, the original problem of computing **A** involved in Theorem 1 is equivalent to finding the eigenvector matrix $𝐌\in {\mathbb{R}}^{t\times q}$, that satisfies $𝐀={𝐔}_{1}PM$, by solving a simpler t×t sized eigenvalue problem on ${\left(\Lambda_{t}+\lambda {𝐈}_{t}\right)}^{-1}{𝐏}^{T}{𝐔}_{1}^{T}{𝐒}_{b}{𝐔}_{1}𝐏$. Let us denote ${\hat{\Sigma }}_{t}=\Lambda_{t}+\lambda {𝐈}_{t}$. Recall that ${𝐒}_{b}=𝐗{YY}^{T}{𝐗}^{T}$ and $q=\text{ rank}({𝐒}_{b}$). Let $𝐁={{\hat{\Sigma }}_{t}}^{-1/2}{𝐏}^{T}{𝐔}_{1}^{T}XY\in {\mathbb{R}}^{t\times k}$, and $𝐁={𝐔}_{B}\Sigma_{B}{𝐕}_{B}^{T}$ be the SVD of **B**, where ${𝐔}_{B}\in {\mathbb{R}}^{t\times t}$ and ${𝐕}_{B}\in {\mathbb{R}}^{k\times k}$ are orthogonal, $\Sigma_{B}=\text{diag}(b_{1},\cdots ,b_{t}in{\mathbb{R}}^{t\times k}$ is diagonal with $b_{1}\geq \cdots \geq b_{q}>0=b_{q+1}=\cdots =b_{t}$. Let ${𝐔}_{q}\in {\mathbb{R}}^{t\times q}$ and ${𝐕}_{q}\in {\mathbb{R}}^{k\times q}$ denote the first *q* columns of ${𝐔}_{B}$ and ${𝐕}_{B}$, and $\Sigma_{q}=\text{diag}(b_{1},\cdots ,b_{q}in{\mathbb{R}}^{q\times q}$, such that **B** =${𝐔}_{q}\Sigma_{q}{𝐕}_{q}^{T}$. We are now ready to introduce an efficient way to compute the top *q* eigenvectors of ${{\hat{\Sigma }}_{t}}^{-1}{𝐏}^{T}{𝐔}_{1}^{T}{𝐒}_{b}{𝐔}_{1}𝐏$ associated with the nonzero eigenvalues, as follows:


$${{\hat{\Sigma }}_{t}}^{-1}{𝐏}^{T}{𝐔}_{1}^{T}{𝐒}_{b}{𝐔}_{1}𝐏={{\hat{\Sigma }}_{t}}^{-1/2}\left({{\hat{\Sigma }}_{t}}^{-1/2}{𝐏}^{T}{𝐔}_{1}^{T}XY\right){\left({{\hat{\Sigma }}_{t}}^{-1/2}{𝐏}^{T}{𝐔}_{1}^{T}XY\right)}^{T}{{\hat{\Sigma }}_{t}}^{1/2}={{\hat{\Sigma }}_{t}}^{-1/2}{BB}^{T}{{\hat{\Sigma }}_{t}}^{1/2}=\left({{\hat{\Sigma }}_{t}}^{-1/2}{𝐔}_{B}\right)\Sigma_{B}\Sigma_{B}^{T}{\left({{\hat{\Sigma }}_{t}}^{-1/2}{𝐔}_{B}\right)}^{-1}$$ (12)

where $\Sigma_{B}\Sigma_{B}^{T}=\text{diag}(\Sigma ,0)$. This suggests that ${{\hat{\Sigma }}_{t}}^{-1/2}{𝐔}_{B}$ diagonalizes ${{\hat{\Sigma }}_{t}}^{-1}{𝐏}^{T}{𝐔}_{1}^{T}{𝐒}_{b}{𝐔}_{1}𝐏$, which is exactly what we need. From (12), we see that only the largest q daigonal entries of $\Sigma_{B}\Sigma_{B}^{T}$ are nonzero, thus we have $𝐌={{\hat{\Sigma }}_{t}}^{-1/2}{𝐔}_{q}$.

Based on our analysis, there are essentially three main steps to get the eigenvectors of $({𝐒}_{t}+\lambda {𝐈}_{d}+\alpha {XLX}^{T}{)}^{+}{𝐒}_{b}$.

1) Compute the SVD of **X** as $𝐗={𝐔}_{1}\Sigma_{t}{𝐕}_{1}^{T}$.
2) Eigen decompose ${\hat{\Sigma }}_{t}$ as ${\hat{\Sigma }}_{t}=𝐏\Lambda_{t}{𝐏}^{T}$, for some α.
3) Compute the SVD of **B** as $𝐁={𝐔}_{q}\Sigma_{q}{𝐕}_{q}^{T}$, for some λ.

Then, it is easy to check that $𝐀={𝐔}_{1}𝐏{{\hat{\Sigma }}_{t}}^{-1/2}{𝐔}_{q}$. By doing so, we can observe that, the nonsingularity of ${𝐒}_{t}+\lambda {𝐈}_{d}+\alpha {XLX}^{T}$ is not required at all. Note that ${𝐒}_{t}+\lambda {𝐈}_{d}+\alpha {XLX}^{T}$ may remain singular when λ=0, but our DGRL still works in such case, without suffering from the SSS problem, thereby extending its applicability. In addition, our three-step strategy enjoys several more important advantages. Specifically, the first step needs to be executed only once relative to the size of the candidate set, as it is independent of the regularization parameters α and λ, thus saving huge amount of computation. The second step runs the eigen analysis to ${\hat{\Sigma }}_{t}$, a tractable matrix with size t×t, where *t*, the rank of **X**, is significantly smaller than *d* for SSS problem. The third step enables us to find **M** quickly by applying the reduced SVD to **B** of much smaller size (i.e., t×k), rather than directly calculating the eigenvectors of the t×t matrix ${{\hat{\Sigma }}_{t}}^{-1}{𝐏}^{T}{𝐔}_{1}^{T}{𝐒}_{b}{𝐔}_{1}𝐏$. Considering rank(**X**) ≤ *n*-1 is typically much greater than the number of classes *k*, this trick leads to a big reduction in terms of the computational cost. When choosing the optimal values for α and λ from given candidate sets, we repeat the computations involved in the second and third steps only. So the cross-validation procedure can be performed efficiently, since we are dealing with matrices ${\hat{\Sigma }}_{t}$ and **B** whose sizes are much smaller than that of $({𝐒}_{t}+\lambda {𝐈}_{d}+\alpha {XLX}^{T}{)}^{+}{𝐒}_{b}$.

From Theorem 1, the optimal solution ${𝐐}^{*}=\hat{𝐐}$ is given by the reduced $QR\text{factorization of}=\hat{𝐐}\hat{𝐑}$. Now, let’s go back and address the issue of computing **W** in (7), which can be calculated more reliably through the following proposition.

*Proposition 2*: Let **X**, **Y**, **A**, and $\hat{𝐑}$ be defined as in Theorem 1. Then,**W** can be finally computed by $𝐖=\hat{𝐑}{𝐀}^{T}XY$.

*Proof*: Please see the detailed proof of Proposition 2 in the supplementary material.

### Linear feature extraction and classification

For any centered input pattern $x\in {\mathbb{R}}^{d}$, one can conduct feature extraction using $\hat{𝐐}$ to produce a compact and discriminant data representation ${\hat{𝐐}}^{T}x$, which is subsequently delivered into the learned liner classifier **W** for multicategory classification. More precisely, the target output for x is calculated as


$$\hat{y}={𝐖}^{T}{\hat{𝐐}}^{T}x={𝐘}^{T}{𝐗}^{T}𝐀{\hat{𝐑}}^{T}{\hat{𝐐}}^{T}x={𝐘}^{T}{𝐗}^{T}{AA}^{T}x$$ (13)

It is interesting and important to see that the above computation does not rely on matrices $\hat{𝐐}$ and $\hat{𝐑}$ ultimately. This implies that the QR factorization of **A** can be avoided, which contributes to further decrease the cost. At last, the predicted class label of x is determined by $\hat{𝒴}=\mathrm{\backslash arg}max_{j\in \left\{1,\cdots ,k\right\}}{\left(\hat{y}\right)}_{j}$, where ${\hat{y}}_{j}$ refers to the *j*th element in $\hat{y}$. In empirical studies, we adopt the 1-nearest neighbor classifier (1NN) to evaluate the final recognition accuracy for fairly comparing.

Let $C_{\lambda }=\{\lambda_{1},\cdots ,\lambda_{\left|C_{\lambda }\right|}\}$ and $C_{\alpha }=\{\alpha_{1},\cdots ,\alpha_{\left|C_{\alpha }\right|}\}$ be the candidate sets for the regularization parameters λ≥0 and α≥0, respectively. In *v*-fold cross validation, we randomly split the input data into *v* subsets or folds of roughly equal size. Then, we reserve one subset to assess our model trained on the remaining *v* −1 subsets. This procedure is repeated *v* times such that each subset is used exactly once for validation. In the *l*th fold, the accuracy for each pair of $\left(\alpha_{i},\lambda_{j}\right),i=1,\cdots ,\left|C_{\alpha }\right|,j=1,\cdots ,\left|C_{\lambda }\right|$, is defined as Acc(*l*, *i*, *j*). Moreover, the average accuracy across all *v* partitions is reported as Acc (*i*, *j*). The optimal values $\alpha_{i^{*}}$ and $\lambda_{j^{*}}$ are the one with ($i^{*}$,$j^{*}$) = arg max _*i*, *j*_ Acc(*i*, *j*). Details of our DGRL model selection algorithm are outlined in Algorithm 1.

Next, analyze the computational complexity of Algorithm 1. Both Lines 8 and 9 take $𝒪(dn^{2})$ time for constructing the graph Laplacian and computing the SVD. To multiply two matrices, Lines 10 and 11 take $𝒪(t^{2}n)$, 𝒪(dnk), and 𝒪(tdk) time, respectively. For each choice $\alpha_{i}$, Line 13 takes $𝒪(tn^{2}+nt^{2})$ time, Lines 14 takes $𝒪(t^{3})$ time to perform eigen-decomposition on a t×t matrix, and Line 15 takes $𝒪(dt^{2}+t^{2}k)$ time. For each choice $\lambda_{j}$, Line 17 takes 𝒪(t) time to invert the diagonal matrix ${\hat{\Sigma }}_{t}$. The SVD computation in Line 18 takes $𝒪(tk^{2})$ time. In Lines 19 and 20, it takes 𝒪(dtq+kdq+ndk+mdk) time for the matrix multiplications, where *q* = rank(**H**_*b*_) is less than or equal to k−1. Line 21 takes $𝒪(n^{2}k)$ time to perform 1NN. Thus, the total cost for estimating the best parameters is about $𝒪(v(dn^{2}+t^{2}n+dnk+tdk+|C_{\alpha }|(tn^{2}+nt^{2}+t^{3}+dt^{2}+t^{2}k+\left|C_{\lambda }\right|(tk^{2}+dtq+kdq+ndk+mdk+n^{2}k))))$. Since t≤min(d,n−1) and *m* < *n*, considering that *d* > *n* and d≫k in small sample size problems, the whole cost is simplified to $𝒪(vdn|C_{\alpha }|(n+\left|C_{\lambda }\right|k))$.

Algorithm 1 Model Selection for DGRL

Input: Data matrix **D** and zero-one label matrix **G**;

Parameters β and *K* in (4); Candiate sets $C_{\lambda }$ and $C_{\alpha }$.

1: **for** *l* = 1 to *v* **do**     // *v* -fold cross validation

2:       Form training set **D**^*l*^ with label matrix **G**^*l*^;

3:       Form validation set ${𝐃}^{\overset{―}{l}}$ with label matrix ${𝐆}^{\overset{―}{l}}$;

4:       *n* = size(**D**^*l*^, 2); *m* = size(${𝐃}^{\overset{―}{l}}$, 2);

5:       Center training matrix **X**^*l*^$\leftarrow {𝐃}^{l}-\frac{1}{n}{𝐃}^{l}1_{n}1_{n}^{T}$;

6:       Center validation matrix ${𝐗}^{\overset{―}{l}}\leftarrow {𝐃}^{\overset{―}{l}}-\frac{1}{n}{𝐃}^{l}1_{n}1_{n}^{T}$;

7:       Normalize training labels **Y**^*l*^ $\leftarrow {𝐆}^{l}(({𝐆}^{l}{)}^{T}{𝐆}^{l}{)}^{-1/2}$;

8:       Form Laplacian matrix **L** from **S** using **X**^*l*^ and **G**^*l*^;

9:       Compute the skinny SVD of **X**^*l*^ =${𝐔}_{1}\Sigma_{t}{𝐕}_{1}^{T}$;

10:      ${𝐇}_{1}\leftarrow \Sigma_{t}{𝐕}_{1}^{T}$; *t*=rank(**X**^*l*^);

11:      ${𝐇}_{b}\leftarrow {𝐗}^{l}{𝐘}^{l};$ ${𝐇}_{c}\leftarrow {𝐔}_{1}^{T}{𝐇}_{b}$;

12:      **for** *i* = 1 to $\left|C_{\alpha }\right|$ **do**         // $\left|C_{\alpha }\right|$ choices for α

13:         ${\tilde{\Sigma }}_{t}\leftarrow {𝐇}_{1}({𝐈}_{n}+\alpha_{i}𝐋mathbfH_{1}^{T}$;

14:         Eigen decompose ${\tilde{\Sigma }}_{t}=𝐏\Lambda_{t}{𝐏}^{T}$;

15:         ${𝐇}_{2}\leftarrow {𝐔}_{1}𝐏$; ${𝐇}_{3}\leftarrow {𝐏}^{T}{𝐇}_{c}$;

16:         **for** *j* = 1 to $\left|C_{\lambda }\right|$ **do**      // $\left|C_{\lambda }\right|$ choices for λ

17:           ${\hat{\Sigma }}_{t}\leftarrow \Lambda_{t}+\lambda_{j}{𝐈}_{t}$; $\leftarrow {{\hat{\Sigma }}_{t}}^{-1/2}{𝐇}_{3}$;

18:           Compute the skinny SVD of **B=**${𝐔}_{q}\Sigma_{q}{𝐕}_{q}^{T}$;

19:           $𝐀\leftarrow {𝐇}_{2}{{\hat{\Sigma }}_{t}}^{-1/2}{𝐔}_{q}$; $\tilde{𝐀}\leftarrow {𝐇}_{b}^{T}{AA}^{T}$;

20:           ${\hat{𝐘}}^{l}\leftarrow {\left(\tilde{𝐀}{𝐗}^{l}\right)}^{T}$; ${\hat{𝐘}}^{\overset{―}{l}}\leftarrow {\left(\tilde{𝐀}{𝐗}^{\overset{―}{l}}\right)}^{T}$;

21:           Run 1NN on (${\hat{𝐘}}^{l},$ ${\hat{𝐘}}^{\overset{―}{l}}$) and compute the validation accuracy,

             denoted as Acc (*l*, *i*, *j*);

22:       end for

23:            end for

24: end for

25: Acc (*i*, *j*)$\begin{array}{c} \leftarrow (1/v)\sum_{l=1}^{v}Acc(l,i,j) \end{array}$;

26: ($i^{*}$,$j^{*}$)$\leftarrow \mathrm{\backslash arg}\max_{i,j}\text{Acc}(i,j)$;

**Output:** The best parameter pair $\left(\alpha_{i^{*}},\lambda_{j^{*}}\right)$.

### Kernel DGRL

In this section, we further generalize DGRL to its nonlinear counterpart by virtue of the kernel trick [6], which enables us to uncover the intrinsic structure of nonlinearly distributed data. Let $\begin{array}{c} \phi :d\in {\mathbb{R}}^{d}⟶\phi \left(d\right)\in ℱ \end{array}$ be a nonlinear mapping from the input space to a high-dimensional feature space. For simplicity, we assume $ℱ\subset {\mathbb{R}}^{D}$, where D is possibly infinite. The core idea behind kernel DGRL (KDGRL) is to jointly learn the discriminant subspace $Q^{\phi }\in {\mathbb{R}}^{D\times s}$ and the linear classifier $W^{\phi }\in {\mathbb{R}}^{s\times k}$ in this new feature space.

Let us first assume that the data matrix in the feature space ℱ is column centered, i.e., $\sum_{i=1}^{n}\phi \left(x_{i}\right)=0$, and denoted by $\mathrm{\backslash Upphi}(X)=[\phi (x_{1}),\cdots ,\phi (x_{n})]\in {\mathbb{R}}^{D\times n}$ The key observation is that the optimal discriminant vectors in ${𝐐}^{\phi }$ can be expressed as a linear combination of the images of training points in ℱ based on the representer theorem [6]. That is ${𝐐}^{\phi }=\Phi (𝐗boldsymbol\Gamma$ for some matrix $\Gamma \in {\mathbb{R}}^{n\times s}$. Let be the kernel matrix with entries, where is a suitable kernel function satisfying Mercer’s condition [6]. Then, the kernelized version of (6) can be written as


$$\underset{{𝐖}^{\phi },\Gamma }{\min}{\left|\left|𝐊\Gamma {𝐖}^{\phi }-𝐘\right|\right|}_{F}^{2}+\lambda {\left|\left|{𝐖}^{\phi }\right|\right|}_{F}^{2}+\alpha tr\left({\left({𝐖}^{\phi }\right)}^{T}\Gamma^{T}𝐊{𝐋}^{\phi }𝐊\Gamma {𝐖}^{\phi }\right)$$

$$\begin{array}{c} s.t.\;{\mathrm{\backslash Upgamma}}^{T}K\mathrm{\backslash Upgamma}=I_{s} \end{array}$$ (14)

where ${𝐋}^{\phi }={𝐂}^{\phi }-{𝐒}^{\phi }\in {\mathbb{R}}^{n\times n}$ is the graph Laplacian matrix, ${𝐂}^{\phi }$ being the diagonal degree matrix of ${𝐒}^{\phi }$ having elements ${𝐂}_{ii}^{\phi }=\sum_{j}S_{ij}^{\phi }$ and the discriminating similarity matrix ${𝐒}^{\phi }$ is analogous to the definition of S in (4) but over mapped patterns $\phi \left(x_{i}\right)$, *i *= 1,⋯, *n*. Note that the construct of ${𝐒}^{\phi }$ in the feature space ℱ, rather than in the input space ${\mathbb{R}}^{d}$ has the advantage that nonlinear relationships between the input data $x_{i}$ can be better expressed.

Setting the partial derivative of the objective function in (14) with respect to $W^{\phi }$ equal to zero, we obtain


$${𝐖}^{\phi }=(\Gamma^{T}({𝐊}^{2}+\lambda 𝐊+\alpha 𝐊{𝐋}^{\phi }𝐊)\Gamma {)}^{-1}\Gamma^{T}KY$$ (15)

Substituting $W^{\phi }$ derived from (15) into (14), we get the following equivalent problem


$$\Gamma^{*}=\underset{\Gamma^{T}𝐊\Gamma ={𝐈}_{s}}{\text{arg max}}\text{ tr}\left((\Gamma^{T}({𝐒}_{t}^{k}+\lambda 𝐊+\alpha 𝐊{𝐋}^{\phi }𝐊)\Gamma {)}^{+}\Gamma^{T}{𝐒}_{b}^{k}\Gamma \right)$$ (16)

where ${𝐒}_{t}^{k}={𝐊}^{2}\in {\mathbb{R}}^{n\times n}$ and ${𝐒}_{b}^{k}=KY{𝐘}^{T}𝐊\in {\mathbb{R}}^{n\times n}$ can be viewed as the total and between-class scatter matrices of **K** when each column in **K** is considered as a data point in ${\mathbb{R}}^{n}$. Furthermore, we also adopt the pseudoinverse to replace the inverse of a matrix in (16) as a new criterion. This allowed our model to work well even if ${𝐒}_{t}^{k}+\lambda 𝐊+\alpha 𝐊{𝐋}^{\phi }𝐊$ is singular.

To get the solution of (16), we need the following theorem.

Theorem 2: Let T be an n × p matrix consisting of the first p eigenvectors of the matrix kt+⁢Skb associated with the nonzero eigenvalues, where $p=\text{rank}(S_{b}^{k}$) and p ≤ k-1. Suppose the eigen-decomposition of $T^{T}KT$ is $T^{T}KT=\hat{U}\hat{\mathrm{\backslash Upsigma}}{\hat{U}}^{T}$ where $\hat{U}\in {\mathbb{R}}^{p\times p}$ is orthogonal, and $\hat{\mathrm{\backslash Upsigma}}\in {\mathbb{R}}^{p\times p}$ is diagonal and positive definite. Then ${\mathrm{\backslash Upgamma}}^{*}=T\hat{U}{\hat{\mathrm{\backslash Upsigma}}}^{1/2}$ solves the maximum problem in (16) with s = p.

Proof: The proof of Theorem 2 is very similar to that of Theorem 1 and can be found in the supplementary file.

### Model selection for KDGRL

Directly applying the eigen decomposition to the n × n sized dense matrix kt+⁢Skb can incur heavy computational overheads especially for large-scale problems. In what follows, we show how to simplify the matrix computations involved in this procedure, thus speeding up the model selection process for KDGRL. Let r be the rank of K and $K=U_{K}{\mathrm{\backslash Upsigma}}_{k}U_{K}^{T}=U_{r}{\mathrm{\backslash Upsigma}}_{r}U_{r}^{T}$ be the SVD of K, where $U_{K}=[U_{r},U_{r}^{⊥}]\in {\mathbb{R}}^{n\times n}$ is orthogonal, ${\mathrm{\backslash Upsigma}}_{K}=\text{ diag}({\mathrm{\backslash Upsigma}}_{r},0in{\mathbb{R}}^{n\times n}$, ${\mathrm{\backslash Upsigma}}_{r}\in {\mathbb{R}}^{r\times r}$ is diagonal and invertible, $U_{r}\in {\mathbb{R}}^{n\times r}$ contains the first r columns of $U_{K}$, and $U_{r}^{⊥}\in {\mathbb{R}}^{n\times (n-r)}$ is the orthogonal complement of $U_{r}$. Let us define ${\tilde{\mathrm{\backslash Upsigma}}}_{r}={\mathrm{\backslash Upsigma}}_{r}^{2}+\lambda {\mathrm{\backslash Upsigma}}_{r}+\alpha {\mathrm{\backslash Upsigma}}_{r}U_{r}^{T}L^{\phi }U_{r}{\mathrm{\backslash Upsigma}}_{r}$. It is easy to verify that ${\tilde{\mathrm{\backslash Upsigma}}}_{r}\in {\mathbb{R}}^{r\times r}$ must be positive definite for arbitrary α=0 and λ=0. Let ${\tilde{\mathrm{\backslash Upsigma}}}_{r}=P_{r}{\mathrm{\backslash Uplambda}}_{r}P_{r}^{T}$ be the eigendecomposition of ${\tilde{\mathrm{\backslash Upsigma}}}_{r}$, where $P_{r}\in {\mathbb{R}}^{r\times r}$ is orthogonal, and ${\mathrm{\backslash Uplambda}}_{r}\in R^{r\times r}$ is diagonal with positive diagonal entries. Then we have


$$\begin{array}{c} ({𝐒}_{t}^{k}+\lambda 𝐊+\alpha 𝐊{𝐋}^{\phi }𝐊{)}^{+}{𝐒}_{b}^{k}={𝐔}_{K}{\left(\Sigma_{K}^{2}+\lambda \Sigma_{K}+\alpha \Sigma_{K}{𝐔}_{K}^{T}{𝐋}^{\phi }{𝐔}_{K}\Sigma_{K}\right)}^{+}{𝐔}_{K}^{T}{𝐒}_{b}^{k}\\ ={𝐔}_{r}{\left(\Sigma_{r}^{2}+\lambda \Sigma_{r}+\alpha \Sigma_{r}{𝐔}_{r}^{T}{𝐋}^{\phi }{𝐔}_{r}\Sigma_{r}\right)}^{-1}{𝐔}_{r}^{T}{𝐒}_{b}^{k}={𝐔}_{r}{𝐏}_{r}\Lambda_{r}^{-1}{𝐏}_{r}^{T}{𝐔}_{r}^{T}{𝐒}_{b}^{k} \end{array}$$ (17)

To decrease the computing cost of the eigenvector matrix T stated in Theorem 2, we establish the following proposition.

Proposition 3:

Suppose the eigenvector of $({𝐒}_{t}^{k}+\lambda 𝐊+\alpha 𝐊{𝐋}^{\phi }𝐊{)}^{+}{𝐒}_{b}^{k}$ is **t** with the nonzero eigenvalue *η*, then $𝐭={𝐔}_{r}{𝐏}_{r}𝐳$, where **z** is an eigenvector of $\Lambda_{r}^{-1}{𝐏}_{r}^{T}{𝐔}_{r}^{T}{𝐒}_{b}^{k}{𝐔}_{r}{𝐏}_{r}$.

Proof: Since $({𝐒}_{t}^{k}+\lambda 𝐊+\alpha 𝐊{𝐋}^{\phi }𝐊{)}^{+}{𝐒}_{b}^{k}𝐭=\eta 𝐭$ and η≠0, according to (17), we obtain$=\frac{1}{\eta }{𝐔}_{r}{𝐏}_{r}\Lambda_{r}^{-1}{𝐏}_{r}^{T}{𝐔}_{r}^{T}{𝐒}_{b}^{k}𝐭={𝐔}_{r}{𝐏}_{r}𝐳,$ for some z. Now, we have to prove z be an eigenvector of ${\mathrm{\backslash Uplambda}}_{r}^{-1}P_{r}^{T}U_{r}^{T}S_{b}^{k}U_{r}P_{r}$. Left-multiplying both sides of equation $U_{r}P_{r}{\mathrm{\backslash Uplambda}}_{r}^{-1}P_{r}^{T}U_{r}^{T}S_{b}^{k}t=\eta t$ by $P_{r}^{T}U_{r}^{T}$ gives ${\mathrm{\backslash Uplambda}}_{r}^{-1}P_{r}^{T}U_{r}^{T}S_{b}^{k}\left(U_{r}P_{r}z\right)=\eta P_{r}^{T}U_{r}^{T}\left(U_{r}P_{r}z\right)=\eta z$

This completes the proof of the proposition.

We proceed by diagonalizing $\Lambda_{r}^{-1}{𝐏}_{r}^{T}{𝐔}_{r}^{T}{𝐒}_{b}^{k}{𝐔}_{r}{𝐏}_{r}$, a tractable matrix with size r×r. Recalling ${𝐒}_{b}^{k}=KY{𝐘}^{T}𝐊$, let us form an r×k matrix $𝐅=\Lambda_{r}^{-1/2}{𝐏}_{r}^{T}{𝐔}_{r}^{T}KY$. Let the SVD of **F** be $𝐅={𝐔}_{F}\Sigma_{F}{𝐕}_{F}^{T}={𝐔}_{P}\Sigma_{P}{𝐕}_{P}^{T}$, where ${𝐔}_{F}\in {\mathbb{R}}^{r\times r}$ and ${𝐕}_{F}\in {\mathbb{R}}^{k\times k}$ are orthogonal, $\Sigma_{F}=\text{diag}(\Sigma_{P},0$)$\in {\mathbb{R}}^{r\times k}$, $\Sigma_{P}\in {\mathbb{R}}^{p\times p}$is diagonal and nonsingular with $p=\text{ rank}({𝐒}_{b}^{k})$, ${𝐔}_{P}\in {\mathbb{R}}^{r\times p}$ and ${𝐕}_{p}\in {\mathbb{R}}^{k\times p}$ consist of the first p columns of ${𝐔}_{F}$and ${𝐕}_{F}$, respectively. It follows that


$$\Lambda_{r}^{-1}{𝐏}_{r}^{T}{𝐔}_{r}^{T}{𝐒}_{b}^{k}{𝐔}_{r}{𝐏}_{r}=\Lambda_{r}^{-1/2}\left(\Lambda_{r}^{-1/2}{𝐏}_{r}^{T}{𝐔}_{r}^{T}KY\right){\left(\Lambda_{r}^{-1/2}{𝐏}_{r}^{T}{𝐔}_{r}^{T}KY\right)}^{T}\Lambda_{r}^{1/2}=\Lambda_{r}^{-1/2}𝐅{𝐅}^{T}\Lambda_{r}^{1/2}=\left(\Lambda_{r}^{-1/2}{𝐔}_{F}\right)\Sigma_{F}\Sigma_{F}^{T}{\left(\Lambda_{r}^{-1/2}{𝐔}_{F}\right)}^{-1}$$ (18)

where ${\mathrm{\backslash Upsigma}}_{F}{\mathrm{\backslash Upsigma}}_{F}^{T}=\text{diag}({\mathrm{\backslash Upsigma}}_{p}^{2},0)$. It clearly shows that the leftmost p columns of ${\mathrm{\backslash Uplambda}}_{r}^{-1/2}U_{F}$ are all we need, as they form the eigenvectors corresponding to the nonzero eigenvalues of ${\mathrm{\backslash Uplambda}}_{r}^{-1}P_{r}^{T}U_{r}^{T}S_{b}^{k}U_{r}P_{r}$. The above computation is more efficient than applying an eigen-decomposition to ${\mathrm{\backslash Uplambda}}_{r}^{-1}P_{r}^{T}U_{r}^{T}S_{b}^{k}U_{r}P_{r}$ directly, since we work on a much smaller matrix F of size r × k, where, usually, k≪r. By Proposition 3, with the calculated $U_{r}$ and $P_{r}$,we can get $𝐓=U_{r}P_{r}{\mathrm{\backslash Uplambda}}_{r}^{-1/2}U_{p}$. It should be emphasized that computing $U_{r}$ is independent of the regularization parameters, i.e., regardless of the sizes of the candidate sets for λ and α.

The above discussion leads to a three-step procedure for solving the eigenvalue problem on St^k+λK+αKLϕK+⁢Skb

1) Compute the skinny SVD of K as $𝐊={𝐔}_{r}\Sigma_{r}{𝐔}_{r}^{T}$.
2) Eigen decompose ${\tilde{\Sigma }}_{r}={𝐏}_{r}\Lambda_{r}{𝐏}_{r}^{T}$, for given(λ,α).
3) Compute the skinny SVD of F as $F=U_{P}{\mathrm{\backslash Upsigma}}_{p}V_{P}^{T}$.

After determining T, the optimal solution of (16) is obtained from $\Gamma^{*}=T\hat{𝐔}{\hat{\Sigma }}^{-1/2}$ by solving the eigenvalue problem of $T^{T}𝐊T=\hat{𝐔}\hat{\Sigma }{\hat{𝐔}}^{T}$ using Theorem 2. Now, let’s look back at (15) and offer an efficient way for computing ${𝐖}^{\phi }$ without matrix inversion. The proposition below illustrates this.

Proposition 4: Let K, Y, $\hat{𝐔}$, and $\hat{\Sigma }$ be defined as in Theorem 2. Then, ${𝐖}^{\phi }$ can be given by ${𝐖}^{\phi }={\hat{\Sigma }}^{1/2}{\hat{𝐔}}^{T}T^{T}KY$.

Proof: The detailed proof for Proposition 4 can be found in the supplementary material.

### Nonlinear feature extraction and classification

Once $\Gamma^{*}$ and ${𝐖}^{\phi }$ are computed, for any centered feature mapping $\begin{array}{c} \phi \left(x\right)\in {\mathbb{R}}^{D} \end{array}$, its projection by ${𝐐}^{\phi }$ can then be carried out as $({𝐐}^{\phi }{)}^{T}\phi \left(x\right)=(\Gamma^{*}{)}^{T}{𝐤}_{x}$, where ${𝐤}_{x}=\Phi {\left(𝐗\right)}^{T}\phi \left(x\right)=[k\left(x_{1},x\right),\cdots ,k\left(x_{n},x\right){]}^{T}$ is a n×1 kernel vector. Meanwhile, we perform final classification on discriminant subspace ${𝐐}^{\phi }$ via linear classifier ${𝐖}^{\phi }$. To be more specific, this is done by


$$\hat{y}=({𝐐}^{\phi }{𝐖}^{\phi }{)}^{T}\phi \left(x\right)={𝐘}^{T}{𝐊}^{T}{TT}^{T}{𝐤}_{x}$$ (19)

An interesting observation from (19) is that the calculation of the target output is entirely irrelevant to the matrices $\hat{U}$ and $\hat{\mathrm{\backslash Upsigma}}$. It means that we do not need to compute the eigendecomposition of $T^{T}KT$ at all, thus saving the computational cost further. Finally, the position number with the maximum value of $\hat{y}$ determines the label of x. In our experiments, the 1NN classifier is used for classification as done in DGRL.

In summary, the complete KDGRL model selection algorithm is described in Algorithm 2. The time complexity analysis of Algorithm 2 is as follows: It takes time $𝒪(dn^{2})$ for the formation of kernel matrix and graph Laplacian. Line 9 takes $𝒪(n^{3})$ time for the SVD computation. Lines 10 and 11 take $\left(n^{2}k\right)$, 𝒪(rnk), and $𝒪(r^{2}n+rn^{2})$ time, respectively, for matrix multiplications. For each choice$\alpha_{i}$ and $\lambda_{i}$, it takes $𝒪(r^{3})$ time in Line 16 for the eigen-decomposition. Since ${\hat{\Sigma }}_{r}$ is a diagonal matrix and its inversion cost 𝒪(r), Line 17 takes $𝒪(n^{2}k)$ time. The SVD computation in Line 18 takes $𝒪(rk^{2})$ time. In Lines 19 and 20, it takes $𝒪(nr^{2}+knp+n^{2}k+mnk)$ time for the matrix multiplications, where p = rank($H_{b}^{k}$) is less than or equal to k−1. Line 21 takes $𝒪(n^{2}k)$ time to perform 1NN. Thus, the total cost of our model selection procedure is about $𝒪(v(dn^{2}+n^{3}+n^{2}k+rnk+rn^{2}+|C_{\alpha }|\left|C_{\lambda }\right|(r^{3}+r^{2}k+rk^{2}+nr^{2}+knp+n^{2}k+mnk)))$. Since r<n,k≤r, and m<n, assuming that d>n, then the overall computational complexity can be compacted into $𝒪({vn}^{2}(d+|C_{\alpha }|\left|C_{\lambda }\right|n))$.

Algorithm 2 Model Selection for KDGRL

Input: Data matrix **D** and zero-one label matrix **G**; Kernel function k(·,·);

Parameters β and *K* of ${𝐒}^{\phi }$; Candiate sets $C_{\lambda }$ and $C_{\alpha }$.

1: **for** *l* = 1 to *v* **do**    // *v* -fold cross validation

2:      Form training set **D**^*l*^ with label matrix **G**^*l*^;

3:      Form validation set ${𝐃}^{\overset{―}{l}}$ with label matrix ${𝐆}^{\overset{―}{l}}$;

4:      *n* = size(**D**^*l*^, 2); *m* = size(${𝐃}^{\overset{―}{l}}$, 2);

       // Center the training and validation kernel matrices

5:      **X**^*l*^$\leftarrow \Phi ({𝐃}^{l})-\frac{1}{n}\Phi ({𝐃}^{l}mathbf1_{n}1_{n}^{T}$; $𝐊l\leftarrow ({𝐗}^{l}{)}^{T}{𝐗}^{l}$;

6:      ${𝐗}^{\overset{―}{l}}\leftarrow \Phi \left({𝐃}^{\overset{―}{l}}\right)-\frac{1}{n}\Phi ({𝐃}^{l}mathbf1_{n}1_{m}^{T}$; ${𝐊}^{\overset{―}{l}}\leftarrow {\left({𝐗}^{\overset{―}{l}}\right)}^{T}{𝐗}^{l}$;

7:      Normalize training labels $𝐘l\leftarrow {𝐆}^{l}(({𝐆}^{l}{)}^{T}{𝐆}^{l}{)}^{-1/2}$;

8:      Form Laplacian matrix ${𝐋}^{\phi }$ from ${𝐒}^{\phi }$ using **X**^*l*^ and **G**^*l*^;

9:      Compute the skinny SVD of **K**^*l*^ =${𝐔}_{r}\Sigma_{r}{𝐔}_{r}^{T}$;

10:     ${𝐇}_{b}^{k}\leftarrow {𝐊}^{l}{𝐘}^{l}$; *t*=rank(**K**^*l*^);

11:     ${𝐇}_{c}^{k}\leftarrow {𝐔}_{r}^{T}{𝐇}_{b}^{k};$ ${𝐇}_{1}^{k}\leftarrow \Sigma_{r}{𝐔}_{r}^{T}{𝐋}^{\phi }{𝐔}_{r}\Sigma_{r}$;

12:     **for** *i* = 1 to $\left|C_{\alpha }\right|$ **do**  // $\left|C_{\alpha }\right|$ choices for α

13:        ${𝐇}_{2}^{k}\leftarrow \Sigma_{r}^{2}+\alpha_{i}{𝐇}_{1}^{k}$;

14:        **for** *j*= 1 to $\left|C_{\lambda }\right|$ **do**   // $\left|C_{\lambda }\right|$ choices for λ

15:             ${\tilde{\Sigma }}_{r}\leftarrow {𝐇}_{2}^{k}+\lambda_{j}\Sigma_{r}$;

16:             Eigen decompose ${\tilde{\Sigma }}_{r}={𝐏}_{r}\Lambda_{r}{𝐏}_{r}^{T}$;

17:             $𝐅\leftarrow \Lambda_{r}^{-1/2}{𝐏}_{r}^{T}{𝐇}_{c}^{k}$;

18:            Compute the skinny SVD of 𝐅 **=** ${𝐔}_{p}\Sigma_{p}{𝐕}_{p}^{T}$;

19:          $𝐓\leftarrow {𝐔}_{r}{𝐏}_{r}\Lambda_{r}^{-1/2}{𝐔}_{p}$; $\tilde{𝐓}\leftarrow ({𝐇}_{b}^{K}{)}^{T}{TT}^{T}$;

20:          ${\hat{𝐘}}^{l}\leftarrow {𝐊}^{l}{\tilde{𝐓}}^{T}$; ${\hat{𝐘}}^{\overset{―}{l}}\leftarrow {𝐊}^{\overset{―}{l}}{\tilde{𝐓}}^{T}$;

21:          Run 1NN on (${\hat{𝐘}}^{l},$ ${\hat{𝐘}}^{\overset{―}{l}}$) and compute the

              validation accuracy, denoted as Acc (*l*, *i*, *j*);

22:        end for

23:     end for

24: end for

25: $Acc(i,jleftarrow(1/v)\sum_{l=1}^{v}Acc(l,i,j)$;

26: $\left(i^{*},j^{*}leftarrow\mathrm{\backslash arg}\max_{i,j}\text{Acc}(i,j\right)$;

**Output:** The best parameter pair $\left(\alpha_{i^{*}},\lambda_{j^{*}}\right)$.

## Experiments

In this section, to thoroughly evaluate the performance of DGRL and KDGRL for feature extraction and classification, we conduct extensive experiments on various types of real world applications, involving face, texture, object, and handwritten digit recognition. Several typical relevant works are compared to confirm the superiority of our methods.

### Experimental setup

We have taken six standard and publicly available image databases, namely ORL [27], Extended YaleB [25], CUReT [35], KTH-TIPS [35], COIL-100 [20], and MNIST [36], to support our theoretical results and the effectiveness of the proposed algorithms. Moreover, we carry out comprehensive comparisons of DGRL and KDGRL with some of the state of- the-art methods or related approaches. Among them, LRLR [22], LRRR [22], LRKR and LRKRR [2,24] pertain to the most representative low-rank regression models. RLRLR [17], DLSR [30], ReLSR [31], CLSR [32] and WCSDLSR [37] are currently the leading LSR-based soft label learning methods for multicategory classification problems. DRLPP [38] and LRPER [39] are recently proposed subspace learning method methods. SVM [40] and regularized least squares (RLS) [41] are classical and powerful classifiers. Specifically, DLSR, RLRLR and WCSDLSR introduce a technique called ε-dragging to enlarge the distances of regression targets of different classes. In addition, RLRLR and WCSDLSR constructs a within-class scatter matrix for the relaxed labels to make the projected samples from the same class closer to each other. ReLSR seeks to learn the target matrix directly from data by constraining the margin between the targets of true and false classes of each sample. CLSR is a variant of ReLSR, it considers to reduce the distances of the new learned targets between samples within the same class. RLSL [25] and SALPL [26] are the recent prominent works of representation-based methods which also integrate the least squares loss function into the dimension reduction process to ensure that the extracted features are optimal for recognition. DRLPP uses two transformation matrices to project high-dimensional data into low-dimensional space, which endows it the capability to preserve the local structure of data. LRPER develops a unified framework that integrates LRR, linear regression, and projection learning.

For SVM, classification is performed by employing the LIBSVM Toolbox [40], in which the cost parameter is chosen from {0.001, 0.01, 0.1, 1, 10, 100, 1000}. As special cases of the proposed linear and kernel formulations, LRLR, LRRR, LRKR and LRKRR are implemented by setting the regularization parametersλ and α to particular values as discussed in the following sections. The codes used for DLSR, ReLSR, CLSR, RLSL, RLRLR, SALPL, DRLPP, LRPER and WCSDLSR are released by the corresponding authors, and the suggested parameter settings are adopted from their original articles. The Gaussian RBF kernel is tested in all the kernel-based methods and the kernel width parameter is tuned on the set {0.05, 0.1, 0.5, 1, 1.5, 2, 2.5, 3}. For simplicity, we empirically set *K* = 5 and β=1 for the construction of the similarity matrices defined in our objective functions across all problems except for the ORL case, where the neighborhood size is fixed to 2 owing to the limited training instances per individual during the cross validation procedure. In fact, they could also be further adjusted to refine the results. The major parametersλ and α of our models are both selected from a wide range of {0, 2^−10^, 2^-9.5^,…, 2^3.5^, 2^4^} when performing Algorithms 1 and 2, while the reduced dimension s is kept to *k*-1, where *k* denotes the number of classes. For each database, different number of samples per subject were randomly selected as the training set and the remainders were retained for testing. We search the best parameters of each method by 10-fold cross validation on the training set. Finally, we independently run all the algorithms ten times with the obtained optimal parameters under each experiment and report their mean accuracies as well as standard deviations for fairness of comparison.

### Experiments for face recognition

The ORL and the Extended YaleB databases are widely employed in examining the algorithmic performance on face recognition learning task.

1) ORL Database: The ORL face database contains 40 subjects and each subject comprises 10 face images, which were taken with a tolerance for some tilting and rotation of the face up to 20 degrees. Moreover, some images were captured at different times, with varying lighting, facial expressions, and facial details (e.g., glasses/no glasses).
2) Extended YaleB Database: The Extended YaleB database is composed of 2414 face images from 38 individuals with small variations in facial expressions and head poses. Each individual provides around 64 near frontal images acquired under various controlled illumination conditions.

All of original images in the two face databases were transformed into gray channel and resized to 32 × 32 pixels in the experiments. Then, each image was reshaped to a 1024- dimensional vector for constructing the feature matrix. For ORL, we randomly select 5, 6, 7, and 8 images per object to form the training set, while the test set contains the rest of the images. As to Extended YaleB, we randomly select 15, 20, 25, and 30 images of each individual as training samples, and the remaining images are regarded as test samples. Tables 1 and 2 display the results of all models on ORL and Extended YaleB, respectively, whereby the best classification accuracies in each column are marked by boldface. One can see that with training sets of varying size, both our methods almost always provide better recognition performance than the others. For example, on the ORL database, with 8 images sampled from each object, DGRL achieves more than 1.09% average accuracy scores in comparison with the state-of-the-art LRPER and the most related RLSL, reflecting its outstanding ability to discover the intrinsic discriminative structure of facial data. It is also noted that DGRL delivers comparable results or even better results than RLRLR and WCSDLSR in all the cases. This illustrates that DGRL has better adaptability to different test scenarios.

**Table 1:** Comparison results (mean ± STD %) of different methods on the ORL database.

Methods	5	6	7	8
LRLR	92.65 ± 1.96	94.37 ± 1.38	95.42 ± 2.05	96.25 ± 2.57
LRRR	94.20 ± 1.67	95.63 ± 2.02	96.83 ± 1.46	97.25 ± 1.29
LRKR	94.60 ± 1.39	95.75 ± 1.74	96.67 ± 1.57	97.13 ± 1.32
LRKRR	94.90 ± 1.51	95.69 ± 1.63	96.92 ± 0.97	97.62 ± 1.61
SVM	91.60 ± 1.24	95.19 ± 1.38	95.33 ± 1.97	97.50 ± 1.86
RLS	94.95 ± 1.42	95.69 ± 0.95	96.08 ± 1.57	97.00 ± 2.14
RLRLR	94.65 ± 1.85	96.06 ± 1.64	97.00 ± 1.12	97.75 ± 1.42
DLSR	93.25 ± 2.15	94.87 ± 1.05	96.33 ± 1.68	96.62 ± 1.96
ReLSR	94.30 ± 1.46	96.19 ± 1.08	96.92 ± 1.93	97.08 ± 1.69
CLSR	94.75 ± 1.74	96.25 ± 1.14	97.17 ± 0.90	97.50 ± 2.36
RLSL	93.30 ± 2.35	96.08 ± 1.25	96.13 ± 1.81	97.37 ± 1.38
SALPL	94.25 ± 2.53	96.83 ± 1.88	97.00 ± 1.34	97.75 ± 1.94
WCSDLSR	94.00 ± 1.60	95.75 ± 1.64	96.92 ± 1.47	97.75 ± 1.15
DRLPP	95.45 ± 1.96	96.50 ± 1.84	97.05 ± 2.20	97.83 ± 2.19
LRPER	95.60 ± 1.96	96.63 ± 1.41	97.24 ± 1.29	98.03 ± 1.56
DGRL	**95.65 ± 1.27**	**97.13 ± 1.11**	**98.38 ± 1.04**	**99.12 ± 1.19**
KDGRL	95.35 ± 1.55	96.94 ± 1.08	97.50 ± 1.18	98.63 ± 0.71

**Table 2:** Comparison results (mean ± STD %) of different methods on the extended YaleB database.

Methods	15	20	25	30
LRLR	89.25 ± 0.94	89.89 ± 0.78	82.36 ± 0.99	85.62 ± 0.48
LRRR	91.99 ± 0.83	94.24 ± 0.66	95.75 ± 0.69	96.90 ± 0.62
LRKR	92.62 ± 0.88	95.08 ± 0.54	96.98 ± 0.51	97.10 ± 0.47
LRKRR	93.18 ± 0.68	96.25 ± 0.74	97.64 ± 0.43	98.19 ± 0.42
SVM	87.72 ± 0.91	91.98 ± 0.76	94.60 ± 0.66	96.40 ± 0.55
RLS	92.86 ± 0.95	95.30 ± 0.79	97.12 ± 0.53	97.90 ± 0.48
RLRLR	94.01 ± 1.11	96.14 ± 0.80	97.39 ± 0.64	97.95 ± 0.39
DLSR	91.08 ± 0.79	94.15 ± 1.03	96.30 ± 0.25	96.92 ± 0.74
ReLSR	92.24 ± 0.64	94.58 ± 0.96	96.67 ± 0.65	97.48 ± 0.54
CLSR	93.59 ± 0.95	96.12 ± 0.89	97.30 ± 0.42	98.05 ± 0.41
RLSL	93.70 ± 0.98	95.82 ± 0.49	97.36 ± 0.42	98.17 ± 0.45
SALPL	93.71 ± 1.07	95.99 ± 0.53	97.43 ± 0.58	98.20 ± 0.39
WCSDLSR	94.06 ± 1.02	94.06 ± 1.02	97.49 ± 0.47	98.16 ± 0.45
DRLPP	92.10 ± 1.12	93.01 ± 0.59	94.21 ± 0.62	94.85 ± 0.51
LRPER	93.02 ± 1.01	95.22 ± 0.85	96.82 ± 0.36	97.80 ± 0.47
DGRL	93.86 ± 0.90	96.33 ± 0.56	97.47 ± 0.51	98.26 ± 0.36
KDGRL	**94.45 ± 0.43**	**96.79 ± 0.31**	**98.09 ± 0.35**	**98.67 ± 0.32**

### Experiments for texture recognition

To validate the performance of our proposed methods on the material recognition task, the CUReT and the KTH-TIPS databases were used to conduct experiments.

1) CUReT Database: The CUReT is a classical database for testing and evaluating state-of-the-art texture recognition algorithms. It contains textures from 61 materials, each of which has been imaged under 205 different viewing and illumination conditions. We utilize the same subset of images as [35]. In this commonly used version, each material has 92 images for which a sufficiently large region of texture is visible across all materials.
2) KTH-TIPS Database: The KTH-TIPS database was created to extend CUReT by providing variations in scale in addition to pose and illumination. This makes it particularly popular in the machine learning community. It consists of 10 texture classes, each of which comprises 81 images generated under three viewing angles, three different illumination directions, and nine scales. Comparing to CUReT, KTH-TIPS is a more challenging database for texture image classification and is better suited for testing the new algorithms.

Following the implementation in [35], 1180-dimensional PRICoLBP features are adopted as the image representation of both texture databases to make the results reproducible and comparable. For CUReT, we randomly select 25, 30, 35, and 40 samples per material for training and the remaining samples are used for testing. As to KTH-TIPS, we randomly select 20, 25, 30, and 35 images from each class as training samples and treat the rest of images as test samples. The recognition accuracies of different methods on these two databases are presented in Tables 3 and 4, respectively. It is obvious that KDGRL gets the highest classification rates among all the compared methods in different training and testing conditions, while DGRL is generally better than or comparable to the other competitors in almost all the cases. This illustrates that our methods are feasible to address texture categorization well.

**Table 3:** Comparison results (mean ± STD %) of different methods on the CUReT database.

Methods	25	30	35	40
LRLR	67.42 ± 0.40	79.12 ± 0.56	84.41 ± 0.44	87.60 ± 0.57
LRRR	95.64 ± 0.35	96.26 ± 0.42	96.89 ± 0.50	97.23 ± 0.31
LRKR	95.25 ± 0.31	96.29 ± 0.36	97.01 ± 0.33	97.48 ± 0.36
LRKRR	96.21 ± 0.41	97.08 ± 0.39	97.41 ± 0.31	97.80 ± 0.29
SVM	96.04 ± 0.37	96.80 ± 0.26	97.45 ± 0.39	97.63 ± 0.47
RLS	89.96 ± 0.84	90.86 ± 0.54	91.86 ± 0.42	92.24 ± 0.45
RLRLR	93.30 ± 0.45	94.23 ± 0.54	95.14 ± 0.35	95.56 ± 0.42
DLSR	93.29 ± 0.42	94.42 ± 0.45	94.79 ± 0.34	95.30 ± 0.45
ReLSR	94.53 ± 0.33	95.22 ± 0.35	95.68 ± 0.41	96.03 ± 0.40
CLSR	94.63 ± 0.45	95.51 ± 0.28	96.05 ± 0.44	96.32 ± 0.28
RLSL	95.53 ± 0.64	96.29 ± 0.35	96.78 ± 0.35	97.33 ± 0.24
SALPL	96.18 ± 0.44	97.09 ± 0.29	97.18 ± 0.37	97.67 ± 0.35
WCSDLSR	96.35 ± 0.53	96.91 ± 0.33	97.59 ± 0.27	97.93 ± 0.25
DRLPP	91.69 ± 0.58	92.58 ± 0.56	93.08 ± 0.33	93.81 ± 0.33
LRPER	95.73 ± 0.36	96.59 ± 0.31	97.16 ± 0.20	97.55 ± 0.21
DGRL	96.43 ± 0.41	97.18 ± 0.45	97.54 ± 0.36	97.78 ± 0.21
KDGRL	**96.91 ± 0.35**	**97.39 ± 0.27**	**97.64 ± 0.31**	**98.30 ± 0.23**

**Table 4:** Comparison results (mean ± STD %) of different methods on the KTH-TIPS database.

Methods	20	25	30	35
LRLR	88.39 ± 2.30	89.86 ± 1.83	90.25 ± 1.54	91.41 ± 2.11
LRRR	90.03 ± 2.26	91.04 ± 1.24	92.96 ± 2.34	94.09 ± 1.64
LRKR	90.21 ± 3.34	92.25 ± 2.33	93.69 ± 3.20	95.46 ± 1.52
LRKRR	92.08 ± 1.86	94.18 ± 1.54	95.18 ± 1.33	95.87 ± 1.03
SVM	91.20 ± 2.28	93.52 ± 1.13	95.06 ± 1.26	96.26 ± 1.07
RLS	90.44 ± 1.13	93.02 ± 1.85	94.78 ± 1.18	95.67 ± 1.16
RLRLR	91.11 ± 1.59	93.55 ± 1.25	94.88 ± 1.40	95.57 ± 1.08
DLSR	91.05 ± 1.27	93.07 ± 1.99	94.84 ± 0.97	95.76 ± 1.78
ReLSR	91.26 ± 1.81	93.61 ± 1.52	94.96 ± 1.06	95.80 ± 1.38
CLSR	92.38 ± 0.97	94.00 ± 1.49	95.29 ± 1.39	95.85 ± 1.47
RLSL	91.38 ± 1.44	92.11 ± 1.85	93.82 ± 2.02	94.57 ± 1.56
SALPL	91.98 ± 1.05	93.54 ± 1.60	94.25 ± 1.76	96.11 ± 0.96
WCSDLSR	91.51 ± 1.74	93.64 ± 1.61	94.88 ± 1.26	95.96 ± 1.34
DRLPP	90.56 ± 1.48	92.64 ± 1.51	93.53 ± 1.11	94.02 ± 0.92
LRPER	91.84 ± 1.71	93.46 ± 1.81	94.75 ± 0.90	95.54 ± 1.20
DGRL	92.92 ± 1.01	94.52 ± 0.75	95.67 ± 0.94	96.96 ± 0.66
KDGRL	**93.13 ± 1.39**	**95.29 ± 1.66**	**96.14 ± 0.92**	**97.13 ± 0.98**

### Experiments for object recognition

In this part, we utilize the frequently used famous Columbia Object Image Library (COIL-100) database to test our methods for object recognition task. It includes a total of 7200 images of various views from 100 objects with different lighting conditions. Each object contributes exactly 72 images, which are taken at pose intervals of 5 degrees. All images for our experiments on this database have been cropped and converted to gray-scale images with the size of 32×32 pixels. By doing so, a 1024-dimensional feature representation for each image can be obtained. Furthermore, we randomly select 15, 20, 25, and 30 images of each object as training samples, and the remaining images are taken as test samples.

Table 5 shows the performance comparisons between the proposed methods and these competing ones under different random splits of training and test data. As observed from these experimental results, our two models once again outperform other comparisons with higher accuracies in all the cases, especially when the number of training samples is very small (e.g., 15 images per object), which is more difficult. This clearly states that DGRL and KDGRL also have greater potential in solving the multiclass object recognition problem.

**Table 5:** Comparison results (mean ± STD%) of different methods on the COIL-100 database.

Methods	15	20	25	30
LRLR	71.01 ± 0.80	76.83 ± 0.55	76.85 ± 0.82	81.28 ± 0.67
LRRR	85.34 ± 0.65	89.21 ± 0.35	91.33 ± 0.36	92.81 ± 0.56
LRKR	82.32 ± 0.34	87.08 ± 0.32	89.67 ± 0.54	91.87 ± 0.44
LRKRR	89.08 ± 0.64	92.55 ± 0.46	94.23 ± 0.25	95.72 ± 0.25
SVM	84.81 ± 0.58	88.14 ± 0.51	90.66 ± 0.49	92.46 ± 0.47
RLS	70.82 ± 0.52	73.30 ± 0.47	75.10 ± 0.27	76.55 ± 0.56
RLRLR	81.29 ± 0.89	84.37 ± 0.53	86.48 ± 0.45	87.82 ± 0.33
DLSR	81.69 ± 0.75	84.39 ± 0.33	86.26 ± 0.39	87.44 ± 0.38
ReLSR	82.74 ± 0.51	85.42 ± 0.59	87.39 ± 0.54	89.06 ± 0.63
CLSR	83.05 ± 0.57	85.64 ± 0.28	87.91 ± 0.52	89.21 ± 0.64
RLSL	91.89 ± 0.68	93.87 ± 0.57	94.85 ± 0.44	95.69 ± 0.36
SALPL	86.05 ± 0.55	89.41 ± 0.56	91.73 ± 0.40	93.21 ± 0.44
WCSDLSR	90.60 ± 0.61	91.76 ± 0.47	93.94 ± 0.24	95.42 ± 0.18
DRLPP	89.51 ± 0.36	90.55 ± 0.39	92.53 ± 0.35	94.58 ± 0.33
LRPER	91.13 ± 0.69	92.88 ± 0.52	94.37 ± 0.42	95.91 ± 0.65
DGRL	91.92 ± 0.59	93.99 ± 0.35	95.41 ± 0.34	96.33 ± 0.30
KDGRL	**92.23 ± 0.50**	**94.37 ± 0.36**	**95.77 ± 0.20**	**96.76 ± 0.32**

### Experiments for handwritten digit recognition

In the end, we ran experiments on the MNIST database to verify the performance of the proposed methods in handwritten digit recognition. The entire MNIST database covers 60000 training images and 10000 test images falling into 10 categories from “0” to “9”. Each digit image is of size 28×28 pixels, with 256 gray levels per pixel. This results in a 784-dimensional feature vector, which we use to describe each image. As in [36], we select the first 2000 instances from 60000 training images to make up our training set and take the first 2000 instances from 10000 test images as our test set. Thus, there are about 200 images of each digit in both the training and test sets. A random split with 30, 60, 90, and 120 images per category from training set are selected for training.

The evaluation results are listed in Table 6, from which we can find that KDGRL still consistently produces the best testing accuracy among comparison counterparts in different kinds of data splits. At the same time, DGRL yields a better recognition performance than those of linear algorithms, such as RLS, RLRLR, LRLR, LRRR, DLSR, ReLSR, CLSR, RLSL, SALPL, WCSDLSR, DRLPP and LRPER, but fails short in contrast to all the kernel-based methods, including LRKR, LRKRR, SVM, and KDGRL. The reason may be that the distribution of handwritten digit patterns is highly sparse and complex, whereas DGRL cannot adequately handle such inherent nonlinear problem due to its linear nature, in particular with the case of insufficient training data. Actually, all the other linear methods compared on this subset encounter the same issue as in DGRL. Comparatively, after exploiting the RBF kernel to map the original handwritten digit patterns into an implicit high-dimensional feature space, the discriminability of KDGRL is dramatically enhanced, resulting in a significant improvement in this task.

**Table 6:** Comparison results (mean ± STD%) of different methods on the MNIST database.

Methods	30	60	90	120
LRLR	49.71 ± 1.30	50.18 ± 2.34	53.55 ± 3.91	62.99 ± 1.35
LRRR	73.01 ± 1.64	75.50 ± 1.00	77.46 ± 0.65	78.41 ± 0.76
LRKR	83.03 ± 0.72	85.95 ± 0.83	87.30 ± 0.77	88.25 ± 0.60
LRKRR	83.50 ± 1.12	87.93 ± 0.78	88.12 ± 0.43	89.65 ± 0.48
SVM	82.63 ± 1.52	86.75 ± 0.66	87.99 ± 0.46	88.82 ± 0.54
RLS	71.12 ± 1.84	74.30 ± 0.92	75.62 ± 1.15	76.37 ± 0.62
RLRLR	77.33 ± 2.30	80.19 ± 0.71	81.03 ± 0.86	82.11 ± 0.80
DLSR	76.67 ± 1.25	78.84 ± 0.92	80.08 ± 0.73	80.82 ± 0.93
ReLSR	76.97 ± 1.05	79.73 ± 1.06	80.65 ± 0.77	81.79 ± 0.85
CLSR	77.35 ± 0.92	80.26 ± 0.98	81.16 ± 0.73	82.20 ± 0.31
RLSL	77.28 ± 1.93	79.28 ± 1.12	80.03 ± 0.77	81.27 ± 0.90
SALPL	73.22 ± 1.65	76.27 ± 1.04	77.67 ± 0.56	78.54 ± 0.69
WCSDLSR	73.12 ± 1.21	75.88 ± 1.76	78.10 ± 1.06	79.66 ± 0.75
DRLPP	72.93 ± 1.76	75.02 ± 1.05	76.37 ± 0.75	78.87 ± 0.62
LRPER	76.11 ± 1.68	79.50 ± 1.30	80.70 ± 0.60	81.03 ± 0.87
DGRL	78.03 ± 1.36	80.54 ± 0.90	81.24 ± 0.66	82.27 ± 0.63
KDGRL	**84.26 ± 0.83**	**88.12 ± 0.51**	**89.50 ± 0.41**	**90.79 ± 0.33**

### Experimental analysis

From Tables 1–6, we can see that the proposed DGRL and KDGRL exhibit favorable recognition performance on four different applications. According to these experimental results, we draw the following observations and conclusions.

First, for all the six databases, DGRL and KDGRL can consistently improve the classification accuracy over LRRR and LRKRR, respectively. Note that, in our experiments, we implement LRRR by setting α=0 in (6) and LRKRR by setting α=0 in (14). This conforms to our motivation of incorporating the local class information to enhance feature extraction and classification. And it empirically proves that the enforced graph regularizer contributes positively in boosting the overall learning performance. On the other hand, low rank regression type of methods (LRLR, LRRR, LRKR, and LRKRR) act as special cases of our framework by using different λ and α. Hence, the proposed formulations are more flexible and expected to outperform, or at least equal to, those approaches when the two regularization parameters are tuned properly with Algorithms 1 and 2. This has been confirmed by our experiments, from another view point, also testify the developed two model selection algorithms are quite successful.

Second, CLSR performs slightly better than DLSR and ReLSR, but worse than our methods in all the cases. This is likely because that in DLSR and ReLSR, the ε –dragging technique or margin constraint used to relax the label matrix will also enlarge the distances of the regression targets between samples from the same class [20], which often degrades the recognition accuracy. What’s more, unduly pursuing the largest margins between classes with the minimum regression loss magnifies the possibility of overfitting. Although CLSR can ensure that the samples in the same class have similar soft target labels, it is directly performed on the original inputs without the dimension reduction step. This may not always be a good choice if the data has very large dimensions, and contains many redundant features even noises which are harmful to the recognition tasks. Intuitively, it is necessary and beneficial to simultaneously perform feature learning and classification so that the two phases can be boosted mutually during training, and thus the overall optimality in algorithmic performance is guaranteed. We can see that our methods not only exploit the latent low-rank subspace to find the compact and discriminative representation of data by modeling the correlations between different samples, but also tend to keep the learned representations sharing the same label close to each other while those with different labels are far apart in each local neighborhood in the target space through the graph structure used in SOLPP, and thereby more in line with the goal for classification.

Third, as the size of the training set increases, the classification performance of our methods improve steadily and trend to be more stable. We attribute this to the fact that a larger set of training data can sample the underlying distribution more accurately than a smaller one. We also notice that our methods provide superior results even under a small training sample setting. For example, on the ORL and KTH-TIPS databases who have a small number of instances in each class, both DGRL and KDGRL consistently beat all the other methods with the desirable recognition rates. These experimental results agree with the previous theoretical analysis and further demonstrate that our algorithms can address small sample size problems very well. Besides, in situations where the number of data points exceeds the dimension of the data space, our approaches still perform comparably well and achieve remarkable testing accuracy. Our experiments on the CUReT and COIL-100 databases nicely bear this out. That is, the effectiveness of the proposed models are not confined to under sampled problems, and they can have wider application scopes in real-world tasks.

Fourth, nonlinear extensions based on kernel learning usually lead to better performance than their linear counterparts. For example, LRKR consistently delivers higher classification accuracy in comparison with LRLR, and the differences are very significant on the Extended YaleB, CUReT, KTH-TIPS, COIL-100 and MNIST databases. The similar observation occurs for the LRKRR and KDGRL as well. For Extended YaleB, KTH-TIPS, COIL-100 and MNIST, LRKRR outperforms LRRR by a large margin. On the whole, of the different databases and different experimental settings, KDGRL holds the best recognition rates among all the competitors except for the OLR case, where the average accuracy of KDGRL would be comparable with that of DGRL. Especially on the MNIST subset, KDGRL yields a considerable performance gain against DGRL in each setting over various training numbers. From these results, we can conclude that using kernel trick is indeed helpful to improve the generalization ability of LRKR, LRKRR, and KDGRL, and it has also been confirmed by other researchers. The essential reason is that the highly nonlinear structure data will be linearized and simplified in the kernel-induced feature space, such that linear techniques can be readily applied to the subsequent data representation and classification.

Finally, our methods always enjoy more promising performances than RLS, RLSL and SALPL on all six databases. Moreover, our methods achieve better recognition performance than RLRLR, WCSDLSR, DRLPP and LRPER in most cases. We confer that for RLSL and SALPL, holding the main energy of the original data by exploring the best reconstruction of the new representation is a good idea to weaken the disturbance of noise or errors from corrupted samples, but this is not sufficient to guarantee the extracted features have the most discriminate power for classification. Not to mention that they lose sight of the locality and neighborhood properties of data. RLS, WCSDLSR and LRPER overlook the local structure of data. Generally, local structure also contains lots of discriminative information. Besides, RLS, RLRLR, DLSR, ReLSR, CLSR and WCSDLSR only uses a single matrix to transform samples into the target space. However, such a single transformation has less freedom to obtain better margins. DRLPP is an unsupervised algorithm, in which label information does not take into consideration. However, the underlying class information is crucial for classification. By contrast, in our proposed models, both global and local information within data as well as the underlying discriminative knowledge are naturally coded into a unified framework by means of low-rank and graph embedding, which enables the learned projection to capture the intrinsic and discriminant structures reside in data so as to further improve the recognition accuracy. In particular, in most cases, the results of DGRL are even better than some kernel-based nonlinear approaches (e.g., LRKR and LRKRR), which only disclose the global correlation relationships of classes. This indicates the advantages of imposing the inherent local manifold geometric structure in a supervised manner to complement the single global Euclidean structure in the representation. Aside from that, RLSL, SALPL, RLRLR, WCSDLSR, DRLPP, and LRPER adopt alternating iterative optimization strategies to solve their non-convex objective functions and can easily converge to a local minimum, inducing inferior results. On the contrary, both problems (6) and (14) are all convex, they have analytic solutions, which makes DGRL and KDGRL simpler and more stable, encouraging a satisfactory performance on classification.

### Parameter sensitivity analysis

**Fig 1:**
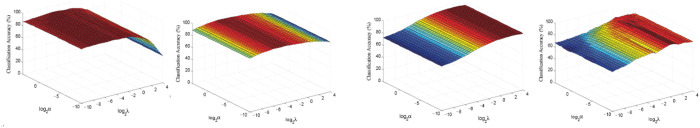
Parameter selection for DGRL on validation set with respect to different values of λ and α on the (a) Extended YaleB database (# Tr = 15), (b)CUReT database (# Tr = 25), (c) COIL-100 database (# Tr = 25), and (d) MNIST database (# Tr = 30), respectively.

**Fig 2:**
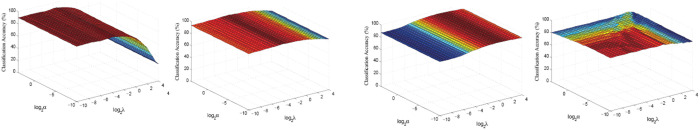
Parameter selection for KDGRL on validation set with respect to different values of λ and α on the (a) Extended YaleB database (# Tr = 15), (b)CUReT database (# Tr = 25), (c) COIL-100 database (# Tr = 25), and (d) MNIST database (# Tr = 30), respectively.

In this study, we conduct experiments on the Extended YaleB, CUReT, COIL-100, and MNIST databases to examine how the proposed model selection algorithms work in practice. Figs 1 and 2 respectively depict parameter estimation results of DGRL and KDGRL on these four databases, where #Tr denotes the number of training samples per class selected for evaluation. This intuitively shows the influence of parameters λ and α on classification performance. As can be seen, our two methods perform well over a pretty wide range of parameter settings for different recognition applications. More specifically, both of them are very robust to the changes in value of α. As for λ, it should not be too large or small, and the classification accuracy curve is relatively smooth in some local areas when λ located in a feasible interval. This is mainly because a very large or very small λ may incur either under fitting or overfitting issues in the learned model, since it controls the complexity of the classifier. Overall, our proposed Algorithms 1 and 2 are effective in identifying the best suitable combinations of λ and α for various tasks as long as they are restricted in a reasonable search space.

### Running time comparison

In this section, we empirically compare the computational cost of different algorithms as the size of training set increases on the Extended YaleB database. The experiments are carried out in MATLAB R2017b environment running on an ordinary PC with 3.0-GHz CPU and 16-GB RAM. Table 7 records the average training time (in seconds) across 10 trials of each method under the best recognition performance on different parameters. It is clear from the table that our methods have much lower costs in time than those of RLSL and SALPL. The main reason is that both RLSL and SALPL need to iteratively update subspace and classification model variables until convergence in the training phase while ours give the closed form solutions which can be directly obtained by respectively solving the generalized eigenvalue problems (9) and (16) only once. As expected, RLS, LRRR and LRKRR run fast compared with ours because the graph regularizer requires extra computation. In addition, DLSR, ReLSR, and CLSR gain rapid learning speed when compared to KDGRL, since they merely serve as lazy linear classifiers without nonlinear mapping and feature extraction processes. Interestingly, DGRL is computationally more efficient than SVM, RLRLR and CLSR in this comparison. It is also noted that DGRL and KDGRL deliver comparable results or even better results than all the compared methods (see Table 2). In this sense, our methods achieve quite promising recognition results with acceptable running time.

**Table 7:** Average training time (seconds) of different methods with different size of training set on the Extended YaleB database.

Methods	Average training time (s)			
	15 × 38	25 × 38	30 × 38	35 × 38
LRRR	0.3875	0.6448	1.0552	1.3406
LRKRR	0.3568	0.5486	0.7908	1.0861
SVM	0.6649	1.1016	1.6120	2.1665
RLS	**0.0404**	**0.0632**	**0.0814**	**0.0974**
RLRLR	1.7725	2.3785	2.6741	2.8280
DLSR	0.1288	0.1632	0.2043	0.2563
ReLSR	0.7257	0.9436	1.1871	1.4207
CLSR	2.3642	3.1167	3.9121	4.6758
RLSL	36.3851	38.0355	39.6935	41.5449
SALPL	29.3846	42.9869	60.7456	87.0394
DGRL	0.5646	0.8894	1.3831	1.7340
KDGRL	3.1941	5.6186	9.0359	13.0047

## Conclusion

In this article, we propose a novel DGRL model which explicitly exploits both global and local class information from data so as to learn a more compact and discriminative representation for recognition. DGRL seamlessly integrates feature learning and ridge regression into a unified framework, such that the obtained projecting subspace has more discriminating power, and thus is competent to perform classification tasks. Moreover, a supervised graph constraint is tailored to make the best of the underlying discriminative information characterized by the manifold structure of data, which allows to further enlarge the distances between different classes while simultaneously heighten the coherence of the learned interclass representation in each local neighborhood in the target space. We also extend DGRL to its kernelized version named KDGRL with an implicit kernel mapping to address the highly nonlinear problems. We derived two effective model selection algorithms for DGRL and KDGRL to tune the regularization parameters. Empirical studies have been carried out on six widely used databases, and the experimental results clearly illustrate that our methods are superior to other relevant state of-the-art approaches in terms of classification performance.

In our future work, we plan to extend our models to the semi-supervised discriminative data representation learning scenario by reformulating the regularization term Ψ(*f*) as in [18], where information of the unlabeled samples is encoded into the graph structure. Furthermore, although our methods show remarkable performance when applied to uncontaminated databases, they are sensitive to the data corrupted with severe noises or outliers to some extent, due to the fact that they use the Euclidean distance as the metric. Therefore, imposing robust norms [11], such as the $l_{1}$ or $l_{2,1}$ sparse constraints on both loss function and regularization term to boost the robustness of DGRL and KDGRL needs to be studied further. On the other hand, since KDGRL involves the whole kernel matrix, which is not scalable for large data. Thus, it is worth investigating how to overcome this limitation. Finally, it is interesting to point out that formula (1) is actually a general framework for feature extraction and classification. In addition to the similarity matrix used in (4), several popular criterions, like MFA, LSDA, and LFDA, can also be readily incorporated into our graph regularizer Ψ(*f*) as an alternative of **L**. We will try to systematically compare all possible combinations of these discriminative regularization terms with other types of classifiers or loss functions in the near future.

## Supporting information

S1 Appendix Appendix A: Proof of theorem 1, Appendix B: Proof of Proposition 2, Appendix C: Proof of theorem 2, Appendix D: Proof of Proposition 4. (DOCX)

S1 File Dataset. (ZIP)
