# Supplementary material for: Discriminative graph regularized representation learning for recognition
Source: PLoS One. 2025 Jul 17;20(7):e0326950. doi: 10.1371/journal.pone.0326950 (PMC12270171; doi:10.1371/journal.pone.0326950)
Supplement: S1 Appendix — (DOCX) [file pone.0326950.s001.docx]

**Appendix A: proof of theorem 1**

Before we prove Theorem 1, we first present two lemmas [4].

*Lemma 1*: For any matrix $\mathbf{O}\in\mathbb{R}^{d\times n}$, the following equality holds:${\mathbf{(O}^{T}\mathbf{O)}}^{\boldsymbol{+}}=\mathbf{O}^{\boldsymbol{+}}{\boldsymbol{(}\mathbf{O}^{\boldsymbol{+}}\boldsymbol{)}}^{T}$ .

*Lemma 2*: Let $\mathbf{O}\in\mathbb{R}^{d\times d}$ be symmetric and positive semidefinite and let $\mathbf{u}_{i}$ be the eigenvector of **O** corresponding to the *i*-th largest eigenvalue $\mu_{i}$. Then, for any $\mathbf{J}\in\mathbb{R}^{d\times s} (s\leq d)$ with orthonormal columns, the following inequality holds

$\mathrm{tr}\left( \mathbf{J}^{T}\mathbf{OJ} \right)\boldsymbol{\leq}\mu_{1}\boldsymbol{+\cdots+}\mu_{s}$,

where the equality holds if $\mathbf{J=[}\mathbf{u}_{1}\boldsymbol{, \cdots,}\mathbf{u}_{s}\mathbf{]}\check{\mathbf{Q}}$, for any orthogonal matrix $\check{\mathbf{Q}}\mathbb{\in R}^{s\times s}$.

The proof of Theorem 1 is essentially similar to that of the Theorem 3.1 in [4], thus we give the following proof sketch for this theorem.

*Proof*: It is easy to check that the null space, **U**_2_ of **X***^T^* is also a subset of the null space of **S***_b_*. That is $\mathbf{U}_{2}^{T}\mathbf{S}_{b}\boldsymbol{=}\mathbf{0}$ and **S***_b_***U**_2_=**0**. It follows that

$$\mathbf{U}_{2}^{T}\mathbf{S}_{b}\mathbf{U}\boldsymbol{=}\binom{\mathbf{U}_{1}^{T}}{\mathbf{U}_{2}^{T}}\mathbf{S}_{b}\left( \mathbf{U}_{1}\mathbf{,}\mathbf{U}_{2} \right)\mathbf{=}\left( \begin{matrix} \mathbf{U}_{1}^{T}\mathbf{S}_{b}\mathbf{U}_{1} & \mathbf{U}_{1}^{T}\mathbf{S}_{b}\mathbf{U}_{2} \\ \mathbf{U}_{2}^{T}\mathbf{S}_{b}\mathbf{U}_{1} & \mathbf{U}_{2}^{T}\mathbf{S}_{b}\mathbf{U}_{2} \end{matrix} \right)\boldsymbol{=}\left( \begin{matrix} \mathbf{U}_{1}^{T}\mathbf{S}_{b}\mathbf{U}_{1} & \mathbf{0} \\ \mathbf{0} & \mathbf{0} \end{matrix} \right)$$

From (12), we have

$$\mathbf{P}^{T}\mathbf{U}_{1}^{T}\mathbf{S}_{b}\mathbf{U}_{1}\mathbf{P}\boldsymbol{=}{\hat{\boldsymbol{\Sigma}}}_{t}^{1/2}\mathbf{U}_{B}\boldsymbol{\Sigma}_{B}\boldsymbol{\Sigma}_{B}^{T}\mathbf{U}_{B}^{T}{\hat{\boldsymbol{\Sigma}}}_{t}^{1/2}$$

Next, we show that the maximum of tr((${\mathbf{Q}^{T}\left( \mathbf{S}_{t}\boldsymbol{+}\lambda\mathbf{I}_{d}\boldsymbol{+}\alpha\mathbf{XL}\mathbf{X}^{T} \right)\mathbf{Q)}}^{\boldsymbol{+}}\mathbf{Q}^{T}\mathbf{S}_{b}\mathbf{Q}$) is achieved by choosing **Q**=**AN**, for any nonsingular $\mathbf{N}\in\mathbb{R}^{q\times q}$. Recall that **A** = $\mathbf{U}_{1}\mathbf{P}{\hat{\boldsymbol{\Sigma}}}_{t}^{-1/2}\mathbf{U}_{q}$, we consider the following two cases.

Case 1: When $\lambda=0$, let **A**_1_ =$\mathbf{U}\left( \begin{matrix} \mathbf{P}{{\hat{\boldsymbol{\Sigma}}}_{t}^{-1/2}\mathbf{U}}_{B} & \mathbf{0} \\ \mathbf{0} & \mathbf{I}_{d-t} \end{matrix} \right)$, we have

$$\mathbf{A}_{1}^{T}\left( \mathbf{X}\mathbf{X}^{T}\mathbf{+}\lambda\mathbf{I}_{d}\boldsymbol{+}\alpha\mathbf{XL}\mathbf{X}^{T} \right)\mathbf{A}_{1}\boldsymbol{=}\left( \begin{matrix} \mathbf{U}_{B}^{T}{\hat{\boldsymbol{\Sigma}}}_{t}^{-1/2}\mathbf{P}^{T} & \mathbf{0} \\ \mathbf{0} & \mathbf{I}_{d-t} \end{matrix} \right)\mathbf{U}^{T}\left( \mathbf{S}_{t}\boldsymbol{+}\lambda\mathbf{I}_{d}\boldsymbol{+}\alpha\mathbf{XL}\mathbf{X}^{T} \right)\mathbf{U}\left( \begin{matrix} \mathbf{P}{{\hat{\boldsymbol{\Sigma}}}_{t}^{-1/2}\mathbf{U}}_{B} & \mathbf{0} \\ \mathbf{0} & \mathbf{I}_{d-t} \end{matrix} \right)$$

$$\boldsymbol{=}\left( \begin{matrix} \mathbf{U}_{B}^{T}{\hat{\boldsymbol{\Sigma}}}_{t}^{-1/2}\mathbf{P}^{T} & \mathbf{0} \\ \mathbf{0} & \mathbf{I}_{d-t} \end{matrix} \right)\mathbf{U}^{T}\mathbf{U}\left( \begin{matrix} {\tilde{\boldsymbol{\Sigma}}}_{t}\mathbf{+}\lambda\mathbf{I}_{t} & \mathbf{0} \\ \mathbf{0} & \lambda\mathbf{I}_{d-t} \end{matrix} \right)\mathbf{U}^{T}\mathbf{U}\left( \begin{matrix} \mathbf{P}{{\hat{\boldsymbol{\Sigma}}}_{t}^{-1/2}\mathbf{U}}_{B} & \mathbf{0} \\ \mathbf{0} & \mathbf{I}_{d-t} \end{matrix} \right)$$

$$\boldsymbol{=}\left( \begin{matrix} \mathbf{U}_{B}^{T}{\hat{\boldsymbol{\Sigma}}}_{t}^{-1/2}\mathbf{P}^{T} & \mathbf{0} \\ \mathbf{0} & \mathbf{I}_{d-t} \end{matrix} \right)\left( \begin{matrix} \mathbf{P(}\boldsymbol{\Lambda}_{t}\mathbf{+}\lambda\mathbf{I}_{t}\boldsymbol{)}\mathbf{P}^{T} & \mathbf{0} \\ \mathbf{0} & \lambda\mathbf{I}_{d-t} \end{matrix} \right)\left( \begin{matrix} \mathbf{P}{{\hat{\boldsymbol{\Sigma}}}_{t}^{-1/2}\mathbf{U}}_{B} & \mathbf{0} \\ \mathbf{0} & \mathbf{I}_{d-t} \end{matrix} \right)$$

$$\boldsymbol{=}\left( \begin{matrix} \mathbf{U}_{B}^{T}{\hat{\boldsymbol{\Sigma}}}_{t}^{-1/2}{\hat{\boldsymbol{\Sigma}}}_{t}{\hat{\boldsymbol{\Sigma}}}_{t}^{-1/2}\mathbf{U}_{B} & \mathbf{0} \\ \mathbf{0} & {\lambda\mathbf{I}}_{d-t} \end{matrix} \right)\boldsymbol{=}\left( \begin{matrix} \mathbf{I}_{t} & \mathbf{0} \\ \mathbf{0} & \mathbf{0} \end{matrix} \right)\boldsymbol{\equiv}\mathbf{D}_{t}$$

and

$$\mathbf{A}_{1}^{T}\left( \mathbf{XY}\mathbf{Y}^{T}\mathbf{X}^{T} \right)\mathbf{A}_{1}\boldsymbol{=}\left( \begin{matrix} \mathbf{U}_{B}^{T}{\hat{\boldsymbol{\Sigma}}}_{t}^{-1/2}\mathbf{P}^{T} & \mathbf{0} \\ \mathbf{0} & \mathbf{I}_{d-t} \end{matrix} \right)\mathbf{U}^{T}\mathbf{S}_{b}\mathbf{U}\left( \begin{matrix} \mathbf{P}{{\hat{\boldsymbol{\Sigma}}}_{t}^{-1/2}\mathbf{U}}_{B} & \mathbf{0} \\ \mathbf{0} & \mathbf{I}_{d-t} \end{matrix} \right)$$

$$\boldsymbol{=}\left( \begin{matrix} \mathbf{U}_{B}^{T}{\hat{\boldsymbol{\Sigma}}}_{t}^{-1/2}\mathbf{P}^{T} & \mathbf{0} \\ \mathbf{0} & \mathbf{I}_{d-t} \end{matrix} \right)\left( \begin{matrix} \mathbf{U}_{1}^{T}\mathbf{S}_{b}\mathbf{U}_{1} & \mathbf{0} \\ \mathbf{0} & \mathbf{0} \end{matrix} \right)\left( \begin{matrix} \mathbf{P}{{\hat{\boldsymbol{\Sigma}}}_{t}^{-1/2}\mathbf{U}}_{B} & \mathbf{0} \\ \mathbf{0} & \mathbf{I}_{d-t} \end{matrix} \right)$$

$$\boldsymbol{=}\left( \begin{matrix} \mathbf{U}_{B}^{T}{\hat{\boldsymbol{\Sigma}}}_{t}^{-1/2}\boldsymbol{(}\mathbf{P}^{T}\mathbf{U}_{1}^{T}\mathbf{S}_{b}\mathbf{U}_{1}\mathbf{P}\boldsymbol{)}{{\hat{\boldsymbol{\Sigma}}}_{t}^{-1/2}\mathbf{U}}_{B} & \mathbf{0} \\ \mathbf{0} & \boldsymbol{0} \end{matrix} \right)$$

$$\boldsymbol{=}\left( \begin{matrix} \mathbf{U}_{B}^{T}{\hat{\boldsymbol{\Sigma}}}_{t}^{-1/2}{\hat{\boldsymbol{\Sigma}}}_{t}^{1/2}\mathbf{U}_{B}\boldsymbol{\Sigma}_{B}\boldsymbol{\Sigma}_{B}^{T}\mathbf{U}_{B}^{T}{\hat{\boldsymbol{\Sigma}}}_{t}^{1/2}{{\hat{\boldsymbol{\Sigma}}}_{t}^{-1/2}\mathbf{U}}_{B} & \mathbf{0} \\ \mathbf{0} & \boldsymbol{0} \end{matrix} \right)\boldsymbol{=}\left( \begin{matrix} \boldsymbol{\Sigma}_{b} & \mathbf{0} \\ \mathbf{0} & \mathbf{0} \end{matrix} \right)\boldsymbol{\equiv}\mathbf{D}_{b}$$

where $\boldsymbol{\Sigma}_{b}\boldsymbol{=}\boldsymbol{\Sigma}_{B}\boldsymbol{\Sigma}_{B}^{T}\boldsymbol{=}diag(b_{1}^{2},\cdots,b_{t}^{2})\in\mathbb{R}^{t\times t}$ is diagonal with $b_{1}\boldsymbol{\geq\cdots\geq}b_{q}\boldsymbol{>}0\boldsymbol{=}b_{q+1}=\boldsymbol{\cdots=}b_{t}$. Hence, the nonsingular matrix $\mathbf{A}_{1}\in\mathbb{R}^{d\times d}$ simultaneously diagonalizes $\mathbf{S}_{t}\boldsymbol{+}\lambda\mathbf{I}_{d}\boldsymbol{+}\alpha\mathbf{XL}\mathbf{X}^{T}$ and $\mathbf{S}_{b}$ in case of $\lambda=0$. In the lower-dimensional subspace resulting from the linear transformation $\mathbf{Q}\in\mathbb{R}^{d\times s}$, $\mathbf{S}_{t}\boldsymbol{+}\lambda\mathbf{I}_{d}\boldsymbol{+}\alpha\mathbf{XL}\mathbf{X}^{T}$ and $\mathbf{S}_{b}$ become

$$\mathbf{S}_{t}^{L}\mathbf{=}\mathbf{Q}^{T}\left( \mathbf{S}_{t}\boldsymbol{+}\lambda\mathbf{I}_{d}\boldsymbol{+}\alpha\mathbf{XL}\mathbf{X}^{T} \right)\mathbf{Q=}\mathbf{Q}^{T}\left( \mathbf{A}_{1}^{-1} \right)^{T}\left( \mathbf{A}_{1}^{T}\left( \mathbf{S}_{t}\boldsymbol{+}\lambda\mathbf{I}_{d}\boldsymbol{+}\alpha\mathbf{XL}\mathbf{X}^{T} \right)\mathbf{A}_{1} \right)\mathbf{A}_{1}^{-1}\mathbf{Q=}{\tilde{\mathbf{Q}}}^{T}\mathbf{D}_{t}\tilde{\mathbf{Q}}$$

$$\mathbf{S}_{b}^{L}\boldsymbol{=}{\mathbf{Q}^{T}\mathbf{S}}_{b}\mathbf{Q=}\mathbf{Q}^{T}\left( \mathbf{A}_{1}^{-1} \right)^{T}\left( \mathbf{A}_{1}^{T}\mathbf{S}_{b}\mathbf{A}_{1} \right)\mathbf{A}_{1}^{-1}\mathbf{Q=}{\tilde{\mathbf{Q}}}^{T}\mathbf{D}_{b}\tilde{\mathbf{Q}}$$

Where $\tilde{\mathbf{Q}}\boldsymbol{=}\mathbf{A}_{1}^{-1}\mathbf{Q}$.

Let $\tilde{\mathbf{Q}}\boldsymbol{=}\left( \begin{matrix} \mathbf{Q}_{1} \\ \mathbf{Q}_{2} \end{matrix} \right)$ be a partition of $\tilde{\mathbf{Q}}$ so that $\mathbf{Q}_{1}\in\mathbb{R}^{t\times s}$ and $\mathbf{Q}_{2}\in\mathbb{R}^{(d-t)\times s}$. It follows that

$\mathbf{S}_{t}^{L}\mathbf{=}{\tilde{\mathbf{Q}}}^{T}\mathbf{D}_{t}\tilde{\mathbf{Q}}\boldsymbol{=}\mathbf{Q}_{1}^{T}\mathbf{Q}_{1}$, $\mathbf{S}_{b}^{L}\boldsymbol{=}{\tilde{\mathbf{Q}}}^{T}\mathbf{D}_{b}\tilde{\mathbf{Q}}\boldsymbol{=}\mathbf{Q}_{1}^{T}{\boldsymbol{\Sigma}_{b}\mathbf{Q}}_{1}$.

Hence

$${F_{1}\mathbf{=}tr((\mathbf{Q}}^{T}{\left( \mathbf{S}_{t}\boldsymbol{+}\lambda\mathbf{I}_{d}\boldsymbol{+}\alpha\mathbf{XL}\mathbf{X}^{T} \right)\mathbf{Q)}}^{\boldsymbol{+}}{\mathbf{Q}^{T}\mathbf{S}}_{b}\mathbf{Q)=}tr({(\mathbf{Q}_{1}^{T}\mathbf{Q}_{1})}^{+}(\mathbf{Q}_{1}^{T}{\boldsymbol{\Sigma}_{b}\mathbf{Q}}_{1}))=tr({(\mathbf{Q}_{1}\mathbf{Q}_{1}^{\boldsymbol{+}})}^{T}\boldsymbol{\Sigma}_{b}(\mathbf{Q}_{1}\mathbf{Q}_{1}^{\boldsymbol{+}}))$$

where the last equality follows from Lemma 1.

Recall that $\boldsymbol{\Sigma}_{b}\boldsymbol{=}diag(b_{1}^{2},\cdots,b_{t}^{2})$, where $b_{1}\boldsymbol{\geq\cdots\geq}b_{q}\boldsymbol{>}0\boldsymbol{=}b_{q+1}=\boldsymbol{\cdots=}b_{t}$. Let $\mathbf{Q}_{1}\boldsymbol{=}\tilde{\mathbf{U}}\left( \begin{matrix} {\bar{\boldsymbol{\Sigma}}}_{\rho} & \mathbf{0} \\ \mathbf{0} & \mathbf{0} \end{matrix} \right){\tilde{\mathbf{V}}}^{T}$ be the SVD of $\mathbf{Q}_{1}$, where $\tilde{\mathbf{U}}$ and $\tilde{\mathbf{V}}$ are orthogonal, ${\bar{\boldsymbol{\Sigma}}}_{\rho}$ is diagonal, and $\rho$ = rank($\mathbf{Q}_{1}$). Then $\mathbf{Q}_{1}^{\boldsymbol{+}}\boldsymbol{=}\tilde{\mathbf{V}}\left( \begin{matrix} {\bar{\boldsymbol{\Sigma}}}_{\rho}^{\boldsymbol{-}1} & \mathbf{0} \\ \mathbf{0} & \mathbf{0} \end{matrix} \right){\tilde{\mathbf{U}}}^{T}$, and $\mathbf{Q}_{1}\mathbf{Q}_{1}^{\boldsymbol{+}}\boldsymbol{=}\tilde{\mathbf{U}}\left( \begin{matrix} \mathbf{I}_{\rho} & \mathbf{0} \\ \mathbf{0} & \mathbf{0} \end{matrix} \right){\tilde{\mathbf{U}}}^{T}$. It follows that

$$F_{1}\mathbf{=}tr(\left( \mathbf{Q}_{1}\mathbf{Q}_{1}^{\boldsymbol{+}} \right)^{T}\boldsymbol{\Sigma}_{b}\left( \mathbf{Q}_{1}\mathbf{Q}_{1}^{\boldsymbol{+}} \right)\mathbf{)=}\mathrm{tr}\left( \tilde{\mathbf{U}}\left( \begin{matrix} \mathbf{I}_{\rho} & \mathbf{0} \\ \mathbf{0} & \mathbf{0} \end{matrix} \right){\tilde{\mathbf{U}}}^{T}\boldsymbol{\Sigma}_{b}\tilde{\mathbf{U}}\left( \begin{matrix} \mathbf{I}_{\rho} & \mathbf{0} \\ \mathbf{0} & \mathbf{0} \end{matrix} \right){\tilde{\mathbf{U}}}^{T} \right)$$

$=tr\left( \left( \begin{matrix} \mathbf{I}_{\rho} & \mathbf{0} \\ \mathbf{0} & \mathbf{0} \end{matrix} \right){\tilde{\mathbf{U}}}^{T}\boldsymbol{\Sigma}_{b}\tilde{\mathbf{U}}\left( \begin{matrix} \mathbf{I}_{\rho} & \mathbf{0} \\ \mathbf{0} & \mathbf{0} \end{matrix} \right) \right)=\mathrm{tr}\left( {{\tilde{\mathbf{U}}}_{\rho}^{T}\boldsymbol{\Sigma}}_{b}{\tilde{\mathbf{U}}}_{\rho} \right){\leq b}_{1}^{2}+\cdots+b_{q}^{2}$.

where ${\tilde{\mathbf{U}}}_{\rho}$ is the matrix consisting of the first $\rho$ columns of $\tilde{\mathbf{U}}$, the third equality follows since $\tilde{\mathbf{U}}$ is orthogonal combining the fact that tr(**OJ**) = tr(**JO**), for any matrices **O** and **J**, and the last inequality follows from Lemma 2. By Lemma 2 again, the above inequality becomes equality, if ${\tilde{\mathbf{U}}}_{\rho}\boldsymbol{=}\left( \begin{matrix} \mathbf{C} \\ \mathbf{O} \end{matrix} \right)$, for any orthogonal $\mathbf{C}\in\mathbb{R}^{q\times q}$, $\rho$ = *q*, and *s* = *q*. Under this choice of ${\tilde{\mathbf{U}}}_{\rho}$, $\mathbf{Q}_{1}\boldsymbol{=}{\tilde{\mathbf{U}}}_{q}{\bar{\boldsymbol{\Sigma}}}_{q}{\tilde{\mathbf{V}}}^{T}\boldsymbol{=}\left( \begin{matrix} \mathbf{C}{\bar{\boldsymbol{\Sigma}}}_{q}{\tilde{\mathbf{V}}}^{T} \\ \mathbf{O} \end{matrix} \right)$. We observe that the maximization of $F_{1}$ is independent of $\mathbf{Q}_{2}$, and simply set it to zero. Therefore, the maximum of $F_{1}$ is attained when $\tilde{\mathbf{Q}}\boldsymbol{=}\left( \begin{matrix} \mathbf{Q}_{1} \\ \mathbf{Q}_{2} \end{matrix} \right)\boldsymbol{=}\left( \begin{matrix} \mathbf{C}{\bar{\boldsymbol{\Sigma}}}_{q}{\tilde{\mathbf{V}}}^{T} \\ \mathbf{O} \end{matrix} \right)$.

Note that the orthogonal matrices $\mathbf{C}$ and $\tilde{\mathbf{V}}$, and the diagonal matrix ${\bar{\boldsymbol{\sum}}}_{q}$ are arbitrary. Hence, **N** = $\mathbf{C}{\bar{\boldsymbol{\sum}}}_{q}{\tilde{\mathbf{V}}}^{T}$ is an arbitrary nonsingular matrix. It follows that **Q** = **A**_1_ $\tilde{\mathbf{Q}}$= **AN**, for an arbitrary nonsingular **N**, maximizes $F_{1}$, where **A** =$\mathbf{U}_{1}\mathbf{P}{{\hat{\boldsymbol{\Sigma}}}_{t}^{-1/2}\mathbf{U}}_{q}$ consists of the first *q* columns of **A**_1_.

Case 2: When $\lambda>0$, $\mathbf{S}_{t}\boldsymbol{+}\lambda\mathbf{I}_{d}\boldsymbol{+}\alpha\mathbf{XL}\mathbf{X}^{T}$ is assumed to be nonsingular, it is symmetric positive definite. According to results from the symmetric definite generalized eigenvalue problem [3], there exists a nonsingular matrix **A**_2_ $\in\mathbb{R}^{d\times d}$such that

$\mathbf{A}_{2}^{T}\left( \mathbf{S}_{t}\boldsymbol{+}\lambda\mathbf{I}_{d}\boldsymbol{+}\alpha\mathbf{XL}\mathbf{X}^{T} \right)\mathbf{A}_{2}\boldsymbol{=}\mathbf{I}_{d}$ and $\mathbf{A}_{2}^{T}\mathbf{S}_{b}\mathbf{A}_{2}\boldsymbol{=}\mathbf{D}_{b}$.

It is easy to check that **A**_2_=$\left( \begin{matrix} \mathbf{P}{{\hat{\boldsymbol{\Sigma}}}_{t}^{-1/2}\mathbf{U}}_{B} & \mathbf{0} \\ \mathbf{0} & \lambda^{-1/2}\mathbf{I}_{d-t} \end{matrix} \right)$. Similarly, in the lower-dimensional subspace resulting from the linear transformation$\mathbf{Q}\in\mathbb{R}^{d\times s}$, $\mathbf{S}_{t}\boldsymbol{+}\lambda\mathbf{I}_{d}\boldsymbol{+}\alpha\mathbf{XL}\mathbf{X}^{T}$ and $\mathbf{S}_{b}$ become

$$\mathbf{S}_{t}^{L}\mathbf{=}\mathbf{Q}^{T}\left( \mathbf{S}_{t}\boldsymbol{+}\lambda\mathbf{I}_{d}\boldsymbol{+}\alpha\mathbf{XL}\mathbf{X}^{T} \right)\mathbf{Q=}\mathbf{Q}^{T}\left( \mathbf{A}_{2}^{-1} \right)^{T}\left( \mathbf{A}_{2}^{T}\left( \mathbf{S}_{t}\boldsymbol{+}\lambda\mathbf{I}_{d}\boldsymbol{+}\alpha\mathbf{XL}\mathbf{X}^{T} \right)\mathbf{A}_{2} \right)\mathbf{A}_{2}^{-1}\mathbf{Q=}{\tilde{\mathbf{Q}}}^{T}\tilde{\mathbf{Q}}$$

$$\mathbf{S}_{b}^{L}\mathbf{=}\mathbf{Q}^{T}\mathbf{S}_{b}\mathbf{Q=}\mathbf{Q}^{T}\left( \mathbf{A}_{2}^{-1} \right)^{T}\left( \mathbf{A}_{2}^{T}\mathbf{S}_{b}\mathbf{A}_{2} \right)\mathbf{A}_{2}^{-1}\mathbf{Q=}{\tilde{\mathbf{Q}}}^{T}\mathbf{D}_{b}\tilde{\mathbf{Q}}$$

where $\tilde{\mathbf{Q}}\boldsymbol{=}\mathbf{A}_{2}^{-1}\mathbf{Q}$

By Lemma 1, we have

$F_{1}\mathbf{=}\mathrm{tr}\left( \mathbf{(Q}^{T}{\mathbf{(}\mathbf{S}_{t}\boldsymbol{+}\lambda\mathbf{I}_{d}\boldsymbol{+}\alpha\mathbf{XL}\mathbf{X}^{T}\mathbf{)Q)}}^{\boldsymbol{+}}\mathbf{Q}^{T}\mathbf{S}_{b}\mathbf{Q} \right)\boldsymbol{=}\mathrm{tr}\left( \left( {\tilde{\mathbf{Q}}}^{T}\tilde{\mathbf{Q}} \right)^{+}\left( {\tilde{\mathbf{Q}}}^{T}\mathbf{D}_{b}\tilde{\mathbf{Q}} \right) \right)=tr\left( \left( \tilde{\mathbf{Q}}{\tilde{\mathbf{Q}}}^{+} \right)^{T}\mathbf{D}_{b}\left( \tilde{\mathbf{Q}}{\tilde{\mathbf{Q}}}^{+} \right) \right)$.

Recall that $\mathbf{D}_{b}\boldsymbol{=}diag(b_{1}^{2},\cdots,b_{t}^{2},0,\cdots,0)$, where $b_{1}\boldsymbol{\geq\cdots\geq}b_{q}\boldsymbol{>}0\boldsymbol{=}b_{q+1}=\boldsymbol{\cdots=}b_{t}$. Again, let $\tilde{\mathbf{Q}}\boldsymbol{=}\tilde{\mathbf{U}}\left( \begin{matrix} {\bar{\boldsymbol{\Sigma}}}_{\rho} & \mathbf{0} \\ \mathbf{0} & \mathbf{0} \end{matrix} \right){\tilde{\mathbf{V}}}^{T}$ be the SVD of $\tilde{\mathbf{Q}}$, where $\tilde{\mathbf{U}}$ and $\tilde{\mathbf{V}}$ are orthogonal, ${\bar{\boldsymbol{\sum}}}_{\rho}$ is diagonal, and $\rho=rank(\tilde{\mathbf{Q}})$. Then, by the definition of pseudoinverse, ${\tilde{\mathbf{Q}}}^{+}\boldsymbol{=}\tilde{\mathbf{V}}\left( \begin{matrix} {\bar{\boldsymbol{\Sigma}}}_{\rho}^{\boldsymbol{-}1} & \mathbf{0} \\ \mathbf{0} & \mathbf{0} \end{matrix} \right){\tilde{\mathbf{U}}}^{T}$, and $\tilde{\mathbf{Q}}{\tilde{\mathbf{Q}}}^{+}\boldsymbol{=}\tilde{\mathbf{U}}\left( \begin{matrix} \mathbf{I}_{\rho} & \mathbf{0} \\ \mathbf{0} & \mathbf{0} \end{matrix} \right){\tilde{\mathbf{U}}}^{T}$. It follows that

$$F_{1}\mathbf{=}\mathrm{tr}\left( \left( \tilde{\mathbf{Q}}{\tilde{\mathbf{Q}}}^{+} \right)^{T}\mathbf{D}_{b}\left( \tilde{\mathbf{Q}}{\tilde{\mathbf{Q}}}^{+} \right) \right)=\mathrm{tr}\left( \tilde{\mathbf{U}}\left( \begin{matrix} \mathbf{I}_{\rho} & \mathbf{0} \\ \mathbf{0} & \mathbf{0} \end{matrix} \right){\tilde{\mathbf{U}}}^{T}\mathbf{D}_{b}\tilde{\mathbf{U}}\left( \begin{matrix} \mathbf{I}_{\rho} & \mathbf{0} \\ \mathbf{0} & \mathbf{0} \end{matrix} \right){\tilde{\mathbf{U}}}^{T} \right)$$

$$=\mathrm{tr}\left( \left( \begin{matrix} \mathbf{I}_{\rho} & \mathbf{0} \\ \mathbf{0} & \mathbf{0} \end{matrix} \right){\tilde{\mathbf{U}}}^{T}\mathbf{D}_{b}\tilde{\mathbf{U}}\left( \begin{matrix} \mathbf{I}_{\rho} & \mathbf{0} \\ \mathbf{0} & \mathbf{0} \end{matrix} \right) \right)=\mathrm{tr}\left( {\tilde{\mathbf{U}}}_{\rho}^{T}\mathbf{D}_{b}{\tilde{\mathbf{U}}}_{\rho} \right){\leq b}_{1}^{2}+\cdots+b_{q}^{2}$$

where ${\tilde{\mathbf{U}}}_{\rho}$ is the matrix consisting of the first $\rho$ columns of $\tilde{\mathbf{U}}$, and the last inequality follows from Lemma 2. By Lemma 2 again, the above inequality becomes equality, if ${\tilde{\mathbf{U}}}_{\rho}\boldsymbol{=}\left( \begin{matrix} \mathbf{C} \\ \mathbf{O} \end{matrix} \right)$, for any orthogonal $\mathbf{C}\in\mathbb{R}^{q\times q}$, $\rho=q$, and *s* = *q*. Under this setting of ${\tilde{\mathbf{U}}}_{\rho}$,

$\tilde{\mathbf{Q}}\boldsymbol{=}{\tilde{\mathbf{U}}}_{q}{\bar{\boldsymbol{\Sigma}}}_{q}{\tilde{\mathbf{V}}}^{T}\boldsymbol{=}\left( \begin{matrix} \mathbf{C}{\bar{\boldsymbol{\Sigma}}}_{q}{\tilde{\mathbf{V}}}^{T} \\ \mathbf{O} \end{matrix} \right)$.

Since the orthogonal matrices $\mathbf{C}$ and $\tilde{\mathbf{V}}$, and the diagonal matrix ${\bar{\boldsymbol{\sum}}}_{q}$ are arbitrary, **N** = $\mathbf{C}{\bar{\boldsymbol{\Sigma}}}_{q}{\tilde{\mathbf{V}}}^{T}$ is an arbitrary nonsingular matrix. It follows that **Q** = **A**_2_$\tilde{\mathbf{Q}}$ = **AN**, for any nonsingular **N**, maximizes *F*_1_, where **A** = $\mathbf{U}_{1}\mathbf{P}{{\hat{\boldsymbol{\Sigma}}}_{t}^{-1/2}\mathbf{U}}_{q}$ consists of the first *q* columns of **A**_2_.

Up until now, we have proved that for any nonsingular matrix **N**, **Q** = **AN** maximizes the *F*_1_ criterion. Let **A** = $\hat{\mathbf{Q}}\hat{\mathbf{R}}$be a QR factorization of **A**, then we can simply choose **N**$\boldsymbol{=}{\hat{\mathbf{R}}}^{-1}$ so that the columns of **Q** = **AN=**$\hat{\mathbf{Q}}$ are orthogonal to each other. Therefore, **Q^*^** =$\hat{\mathbf{Q}}$ solves the optimization problem in (9). This completes the proof of the theorem.

**Appendix B: proof of Proposition 2**

*Proof*: Since **A** = $\mathbf{U}_{1}\mathbf{P}{{\hat{\boldsymbol{\Sigma}}}_{t}^{-1/2}\mathbf{U}}_{q}\boldsymbol{=}\hat{\mathbf{Q}}\hat{\mathbf{R}}$, we have $\hat{\mathbf{Q}}\boldsymbol{=}\mathbf{A}{\hat{\mathbf{R}}}^{-1}\boldsymbol{=}\mathbf{U}_{1}\mathbf{P}{{\hat{\boldsymbol{\Sigma}}}_{t}^{-1/2}\mathbf{U}}_{q}{\hat{\mathbf{R}}}^{-1}$. Then, it follows that

$$\mathbf{W}={\boldsymbol{(}\hat{\mathbf{Q}}}^{T}{\mathbf{(X}\mathbf{X}^{T}\boldsymbol{+}\lambda\mathbf{I}_{d}\boldsymbol{+}\alpha\mathbf{XL}\mathbf{X}^{T}\boldsymbol{)}\hat{\mathbf{Q}}\boldsymbol{)}}^{-1}{\hat{\mathbf{Q}}}^{T}\mathbf{XY}$$

$$\boldsymbol{=}\left( {\boldsymbol{(}{\hat{\mathbf{R}}}^{-1}\boldsymbol{)}}^{T}\mathbf{U}_{q}^{T}{\hat{\boldsymbol{\Sigma}}}_{t}^{-1/2}\mathbf{P}^{T}\mathbf{U}_{1}^{T}\boldsymbol{(}\mathbf{S}_{t}\boldsymbol{+}\lambda\mathbf{I}_{d}\boldsymbol{+}\alpha\mathbf{XL}\mathbf{X}^{T}\boldsymbol{)}\mathbf{U}_{1}\mathbf{P}{{\hat{\boldsymbol{\Sigma}}}_{t}^{-1/2}\mathbf{U}}_{q}{\hat{\mathbf{R}}}^{-1} \right)^{-1}{\hat{\mathbf{Q}}}^{T}\mathbf{XY}$$

$$\boldsymbol{=}\left( {\boldsymbol{(}{\hat{\mathbf{R}}}^{-1}\boldsymbol{)}}^{T}\mathbf{U}_{q}^{T}{\hat{\boldsymbol{\Sigma}}}_{t}^{-1/2}\mathbf{P}^{T}\mathbf{U}_{1}^{T}\mathbf{U}\left( \begin{matrix} {\tilde{\boldsymbol{\Sigma}}}_{t}\boldsymbol{+}\lambda\mathbf{I}_{t} & \mathbf{0} \\ \mathbf{0} & \lambda\mathbf{I}_{d-t} \end{matrix} \right)\mathbf{U}^{T}\mathbf{U}_{1}\mathbf{P}{{\hat{\boldsymbol{\Sigma}}}_{t}^{-1/2}\mathbf{U}}_{q}{\hat{\mathbf{R}}}^{-1} \right)^{-1}{\hat{\mathbf{Q}}}^{T}\mathbf{XY}$$

$$\boldsymbol{=}\left( {\boldsymbol{(}{\hat{\mathbf{R}}}^{-1}\boldsymbol{)}}^{T}\mathbf{U}_{q}^{T}{\hat{\boldsymbol{\Sigma}}}_{t}^{-1/2}\mathbf{P}^{T}\mathbf{U}_{1}^{T}\mathbf{(}\mathbf{U}_{1}\mathbf{,}\mathbf{U}_{2}\mathbf{)}\left( \begin{matrix} {\tilde{\boldsymbol{\Sigma}}}_{t}\boldsymbol{+}\lambda\mathbf{I}_{t} & \mathbf{0} \\ \mathbf{0} & \lambda\mathbf{I}_{d-t} \end{matrix} \right)\left( \begin{matrix} \mathbf{U}_{1}^{T} \\ \mathbf{U}_{2}^{T} \end{matrix} \right)\mathbf{U}_{1}\mathbf{P}{{\hat{\boldsymbol{\Sigma}}}_{t}^{-1/2}\mathbf{U}}_{q}{\hat{\mathbf{R}}}^{-1} \right)^{-1}{\hat{\mathbf{Q}}}^{T}\mathbf{XY}$$

$$\boldsymbol{=}\left( {\boldsymbol{(}{\hat{\mathbf{R}}}^{-1}\boldsymbol{)}}^{T}\mathbf{U}_{q}^{T}{\hat{\boldsymbol{\Sigma}}}_{t}^{-1/2}\mathbf{P}^{T}\boldsymbol{(}\mathbf{I}_{t}\mathbf{, 0)}\left( \begin{matrix} {\tilde{\boldsymbol{\Sigma}}}_{t}\boldsymbol{+}\lambda\mathbf{I}_{t} & \mathbf{0} \\ \mathbf{0} & \lambda\mathbf{I}_{d-t} \end{matrix} \right)\left( \begin{matrix} \mathbf{I}_{t} \\ \mathbf{0} \end{matrix} \right)\mathbf{P}{{\hat{\boldsymbol{\Sigma}}}_{t}^{-1/2}\mathbf{U}}_{q}{\hat{\mathbf{R}}}^{-1} \right)^{-1}{\hat{\mathbf{Q}}}^{T}\mathbf{XY}$$

$$\boldsymbol{=}\left( {\boldsymbol{(}{\hat{\mathbf{R}}}^{-1}\boldsymbol{)}}^{T}\mathbf{U}_{q}^{T}{\hat{\boldsymbol{\Sigma}}}_{t}^{-1/2}\mathbf{P}^{T}\boldsymbol{(}{\tilde{\boldsymbol{\Sigma}}}_{t}\boldsymbol{+}\lambda\mathbf{I}_{t}\mathbf{)P}{{\hat{\boldsymbol{\Sigma}}}_{t}^{-1/2}\mathbf{U}}_{q}{\hat{\mathbf{R}}}^{-1} \right)^{-1}{\hat{\mathbf{Q}}}^{T}\mathbf{XY}$$

$$\boldsymbol{=}\left( {\boldsymbol{(}{\hat{\mathbf{R}}}^{-1}\boldsymbol{)}}^{T}\mathbf{U}_{q}^{T}{\hat{\boldsymbol{\Sigma}}}_{t}^{-1/2}\mathbf{P}^{T}\mathbf{P}\boldsymbol{(}\boldsymbol{\Lambda}_{t}\boldsymbol{+}\lambda\mathbf{I}_{t}\mathbf{)}\mathbf{P}^{T}\mathbf{P}{{\hat{\boldsymbol{\Sigma}}}_{t}^{-1/2}\mathbf{U}}_{q}{\hat{\mathbf{R}}}^{-1} \right)^{-1}{\hat{\mathbf{Q}}}^{T}\mathbf{XY}$$

$$\boldsymbol{=}\left( {\boldsymbol{(}{\hat{\mathbf{R}}}^{-1}\boldsymbol{)}}^{T}\mathbf{U}_{q}^{T}{\hat{\boldsymbol{\Sigma}}}_{t}^{-1/2}\boldsymbol{(}\boldsymbol{\Lambda}_{t}\boldsymbol{+}\lambda\mathbf{I}_{t}\mathbf{)}{{\hat{\boldsymbol{\Sigma}}}_{t}^{-1/2}\mathbf{U}}_{q}{\hat{\mathbf{R}}}^{-1} \right)^{-1}{\hat{\mathbf{Q}}}^{T}\mathbf{XY}$$

$$\boldsymbol{=}\left( {\boldsymbol{(}{\hat{\mathbf{R}}}^{-1}\boldsymbol{)}}^{T}\mathbf{U}_{q}^{T}{\hat{\boldsymbol{\Sigma}}}_{t}^{-1/2}{\hat{\boldsymbol{\Sigma}}}_{t}{{\hat{\boldsymbol{\Sigma}}}_{t}^{-1/2}\mathbf{U}}_{q}{\hat{\mathbf{R}}}^{-1} \right)^{-1}{\hat{\mathbf{Q}}}^{T}\mathbf{XY}$$

$$\boldsymbol{=}\left( {\boldsymbol{(}{\hat{\mathbf{R}}}^{-1}\boldsymbol{)}}^{T}\mathbf{U}_{q}^{T}\mathbf{U}_{q}{\hat{\mathbf{R}}}^{-1} \right)^{-1}{\hat{\mathbf{Q}}}^{T}\mathbf{XY}\boldsymbol{=}\left( {\boldsymbol{(}{\hat{\mathbf{R}}}^{T}\boldsymbol{)}}^{-1}{\hat{\mathbf{R}}}^{-1} \right)^{-1}{\hat{\mathbf{Q}}}^{T}\mathbf{XY}$$

$$\boldsymbol{=}\hat{\mathbf{R}}\boldsymbol{(}{\hat{\mathbf{R}}}^{T}{\hat{\mathbf{Q}}}^{T}\mathbf{)XY}\boldsymbol{=}\hat{\mathbf{R}}\mathbf{A}^{T}\mathbf{XY}$$

**Appendix C: proof of theorem 2**

*Proof*: Since ${\mathbf{K}=\mathbf{U}}_{K}\boldsymbol{\Sigma}_{K}\mathbf{U}_{K}^{T}\boldsymbol{=}\mathbf{U}_{\boldsymbol{r}}\boldsymbol{\Sigma}_{r}\mathbf{U}_{r}^{T}$ and ${\tilde{\boldsymbol{\Sigma}}}_{r}\boldsymbol{=}{\mathbf{P}_{r}\boldsymbol{\Lambda}_{r}\mathbf{P}}_{r}^{T}$, we can decompose $\mathbf{S}_{t}^{k}\boldsymbol{+}\lambda\mathbf{K}\boldsymbol{+}\alpha\mathbf{K}\mathbf{L}^{\phi}\mathbf{K}$ as follows:

$$\mathbf{S}_{t}^{k}\boldsymbol{+}\lambda\mathbf{K}\boldsymbol{+}\alpha\mathbf{K}\mathbf{L}^{\phi}\mathbf{K=}\mathbf{K}^{2}\boldsymbol{+}\lambda\mathbf{K}\boldsymbol{+}\alpha\mathbf{K}\mathbf{L}^{\phi}\mathbf{K=}\mathbf{U}_{K}\boldsymbol{(}\boldsymbol{\Sigma}_{K}^{2}\boldsymbol{+}\lambda\boldsymbol{\Sigma}_{K}\boldsymbol{+}\alpha\boldsymbol{\Sigma}_{K}\mathbf{U}_{K}^{T}\mathbf{L}^{\phi}\mathbf{U}_{K}\boldsymbol{\Sigma}_{K}\mathbf{)}\mathbf{U}_{K}^{T}$$

$$\mathbf{=}\mathbf{U}_{K}\left( \begin{matrix} \boldsymbol{\Sigma}_{r}^{2}\boldsymbol{+}\lambda\boldsymbol{\Sigma}_{r}\boldsymbol{+}\alpha\boldsymbol{\Sigma}_{r}\mathbf{U}_{r}^{T}\mathbf{L}^{\phi}\mathbf{U}_{r}\boldsymbol{\Sigma}_{r} & \mathbf{0} \\ \mathbf{0} & \mathbf{0} \end{matrix} \right)\mathbf{U}_{K}^{T}\mathbf{=}\mathbf{U}_{K}\left( \begin{matrix} {\mathbf{P}_{r}\boldsymbol{\Lambda}_{r}\mathbf{P}}_{r}^{T} & \mathbf{0} \\ \mathbf{0} & \mathbf{0} \end{matrix} \right)\mathbf{U}_{K}^{T}$$

By $\mathbf{F}={\boldsymbol{\Lambda}_{r}^{-1/2}\mathbf{P}}_{r}^{T}\mathbf{U}_{r}^{T}\mathbf{KY}$ and $\mathbf{F}=\mathbf{U}_{F}\boldsymbol{\Sigma}_{F}\mathbf{V}_{F}^{T}\boldsymbol{=}\mathbf{U}_{P}\boldsymbol{\Sigma}_{P}\mathbf{V}_{P}^{T}$, we have $\mathbf{U}_{r}^{T}\mathbf{KY=}\mathbf{P}_{r}\boldsymbol{\Lambda}_{r}^{1/2}\mathbf{U}_{F}\boldsymbol{\Sigma}_{F}\mathbf{V}_{F}^{T}$.

Recall that $\mathbf{U}_{K}\boldsymbol{=[}\mathbf{U}_{\boldsymbol{r}}\boldsymbol{,}\mathbf{U}_{r}^{\perp}\boldsymbol{]}$ and $\mathbf{S}_{b}^{k}=\mathbf{KY}\mathbf{Y}^{T}\mathbf{K}\in\mathbb{R}^{n\times n}$, it is easy to check that $\mathbf{S}_{b}^{k}\mathbf{U}_{r}^{\perp}\boldsymbol{=0}$ and ${\mathbf{(U}_{r}^{\perp}\boldsymbol{)}}^{T}\mathbf{S}_{b}^{k}\boldsymbol{=0}$, then

$$\mathbf{U}_{K}^{T}\mathbf{S}_{b}^{k}\mathbf{U}_{K}\boldsymbol{=}{\boldsymbol{[}\mathbf{U}_{\boldsymbol{r}}\boldsymbol{,}\mathbf{U}_{r}^{\perp}\boldsymbol{]}}^{T}\mathbf{S}_{b}^{k}\left[ \mathbf{U}_{\boldsymbol{r}}\boldsymbol{,}\mathbf{U}_{r}^{\perp} \right]\boldsymbol{=}\left( \begin{matrix} \mathbf{U}_{r}^{T}\mathbf{S}_{b}^{k}\mathbf{U}_{r} & \mathbf{U}_{r}^{T}\mathbf{S}_{b}^{k}\mathbf{U}_{r}^{\perp} \\ {\mathbf{(U}_{r}^{\perp}\boldsymbol{)}}^{T}\mathbf{S}_{b}^{k}\mathbf{U}_{r} & {\mathbf{(U}_{r}^{\perp}\boldsymbol{)}}^{T}\mathbf{S}_{b}^{k}\mathbf{U}_{r}^{\perp} \end{matrix} \right)\boldsymbol{=}\left( \begin{matrix} \mathbf{U}_{r}^{T}\mathbf{S}_{b}^{k}\mathbf{U}_{r} & \mathbf{0} \\ \mathbf{0} & \mathbf{0} \end{matrix} \right)$$

Next, we show that $\boldsymbol{\Gamma=T\Upsilon}$ maximizes $F_{2}\mathbf{=}tr({(\boldsymbol{\Gamma}^{T}(\mathbf{S}_{t}^{k}\boldsymbol{+}\lambda\mathbf{K}\boldsymbol{+}\alpha\mathbf{K}\mathbf{L}^{\phi}\mathbf{K})\boldsymbol{\Gamma})}^{+}\boldsymbol{\Gamma}^{T}\mathbf{S}_{b}^{k}\boldsymbol{\Gamma})$ for an arbitrary nonsingular matrix $\boldsymbol{\Upsilon}\in\mathbb{R}^{p\times p}$, where $\mathbf{T=}\mathbf{U}_{\boldsymbol{r}}\mathbf{P}_{r}\boldsymbol{\Lambda}_{r}^{-1/2}\mathbf{U}_{p}$.

Let $\mathbf{T}_{1}\mathbf{=}\mathbf{U}_{K}\left( \begin{matrix} \mathbf{P}_{r}\boldsymbol{\Lambda}_{r}^{-1/2}\mathbf{U}_{F} & \mathbf{0} \\ \mathbf{0} & \mathbf{I}_{n-r} \end{matrix} \right)$, it goes

$$\mathbf{T}_{1}^{T}\left( \mathbf{K}^{2}\boldsymbol{+}\lambda\mathbf{K}\boldsymbol{+}\alpha\mathbf{K}\mathbf{L}^{\phi}\mathbf{K} \right)\mathbf{T}_{1}\boldsymbol{=}\left( \begin{matrix} \mathbf{U}_{F}^{T}\boldsymbol{\Lambda}_{r}^{-1/2}\mathbf{P}_{r}^{T} & \mathbf{0} \\ \mathbf{0} & \mathbf{I}_{n-r} \end{matrix} \right)\mathbf{U}_{K}^{T}(\mathbf{S}_{t}^{k}\boldsymbol{+}\lambda\mathbf{K}\boldsymbol{+}\alpha\mathbf{K}\mathbf{L}^{\phi}\mathbf{K})\mathbf{U}_{K}\left( \begin{matrix} \mathbf{P}_{r}\boldsymbol{\Lambda}_{r}^{-1/2}\mathbf{U}_{F} & \mathbf{0} \\ \mathbf{0} & \mathbf{I}_{n-r} \end{matrix} \right)$$

$$\boldsymbol{=}\left( \begin{matrix} \mathbf{U}_{F}^{T}\boldsymbol{\Lambda}_{r}^{-1/2}\mathbf{P}_{r}^{T} & \mathbf{0} \\ \mathbf{0} & \mathbf{I}_{n-r} \end{matrix} \right)\left( \begin{matrix} {\mathbf{P}_{r}\boldsymbol{\Lambda}_{r}\mathbf{P}}_{r}^{T} & \mathbf{0} \\ \mathbf{0} & \mathbf{0} \end{matrix} \right)\left( \begin{matrix} \mathbf{P}_{r}\boldsymbol{\Lambda}_{r}^{-1/2}\mathbf{U}_{F} & \mathbf{0} \\ \mathbf{0} & \mathbf{I}_{n-r} \end{matrix} \right)$$

$$\boldsymbol{=}\left( \begin{matrix} {{\mathbf{U}_{F}^{T}\boldsymbol{\Lambda}_{r}^{-1/2}\mathbf{P}_{r}^{T}\mathbf{P}}_{r}\boldsymbol{\Lambda}_{r}\mathbf{P}}_{r}^{T}\mathbf{P}_{r}\boldsymbol{\Lambda}_{r}^{-1/2}\mathbf{U}_{F} & \mathbf{0} \\ \mathbf{0} & \mathbf{0} \end{matrix} \right)\boldsymbol{=}\left( \begin{matrix} \mathbf{I}_{r} & \mathbf{0} \\ \mathbf{0} & \mathbf{0} \end{matrix} \right)\boldsymbol{\equiv}\mathbf{D}_{r}$$

and

$$\mathbf{T}_{1}^{T}\left( \mathbf{KY}\mathbf{Y}^{T}\mathbf{K} \right)\mathbf{T}_{1}\boldsymbol{=}\left( \begin{matrix} \mathbf{U}_{F}^{T}\boldsymbol{\Lambda}_{r}^{-1/2}\mathbf{P}_{r}^{T} & \mathbf{0} \\ \mathbf{0} & \mathbf{I}_{n-r} \end{matrix} \right)\mathbf{U}_{K}^{T}\mathbf{S}_{b}^{k}\mathbf{U}_{K}\left( \begin{matrix} \mathbf{P}_{r}\boldsymbol{\Lambda}_{r}^{-1/2}\mathbf{U}_{F} & \mathbf{0} \\ \mathbf{0} & \mathbf{I}_{n-r} \end{matrix} \right)$$

$$\boldsymbol{=}\left( \begin{matrix} \mathbf{U}_{F}^{T}\boldsymbol{\Lambda}_{r}^{-1/2}\mathbf{P}_{r}^{T} & \mathbf{0} \\ \mathbf{0} & \mathbf{I}_{n-r} \end{matrix} \right)\left( \begin{matrix} \mathbf{U}_{r}^{T}\mathbf{S}_{b}^{k}\mathbf{U}_{r} & \mathbf{0} \\ \mathbf{0} & \mathbf{0} \end{matrix} \right)\left( \begin{matrix} \mathbf{P}_{r}\boldsymbol{\Lambda}_{r}^{-1/2}\mathbf{U}_{F} & \mathbf{0} \\ \mathbf{0} & \mathbf{I}_{n-r} \end{matrix} \right)$$

$$\boldsymbol{=}\left( \begin{matrix} \mathbf{U}_{F}^{T}\boldsymbol{\Lambda}_{r}^{-1/2}\mathbf{P}_{r}^{T} & \mathbf{0} \\ \mathbf{0} & \mathbf{I}_{n-r} \end{matrix} \right)\left( \begin{matrix} \mathbf{(U}_{r}^{T}\mathbf{KY}\boldsymbol{)}{\mathbf{(U}_{r}^{T}\mathbf{KY}\boldsymbol{)}}^{T} & \mathbf{0} \\ \mathbf{0} & \mathbf{0} \end{matrix} \right)\left( \begin{matrix} \mathbf{P}_{r}\boldsymbol{\Lambda}_{r}^{-1/2}\mathbf{U}_{F} & \mathbf{0} \\ \mathbf{0} & \mathbf{I}_{n-r} \end{matrix} \right)$$

$$\boldsymbol{=}\left( \begin{matrix} \mathbf{U}_{F}^{T}\boldsymbol{\Lambda}_{r}^{-1/2}\mathbf{P}_{r}^{T} & \mathbf{0} \\ \mathbf{0} & \mathbf{I}_{n-r} \end{matrix} \right)\left( \begin{matrix} \mathbf{P}_{r}\boldsymbol{\Lambda}_{r}^{1/2}\mathbf{U}_{F}\boldsymbol{\Sigma}_{F}\boldsymbol{\Sigma}_{F}^{T}\mathbf{U}_{F}^{T}\boldsymbol{\Lambda}_{r}^{1/2}\mathbf{P}_{r}^{T} & \mathbf{0} \\ \mathbf{0} & \mathbf{0} \end{matrix} \right)\left( \begin{matrix} \mathbf{P}_{r}\boldsymbol{\Lambda}_{r}^{-1/2}\mathbf{U}_{F} & \mathbf{0} \\ \mathbf{0} & \mathbf{I}_{n-r} \end{matrix} \right)$$

$$\boldsymbol{=}\left( \begin{matrix} \boldsymbol{\Sigma}_{F}\boldsymbol{\Sigma}_{F}^{T} & \mathbf{0} \\ \mathbf{0} & \mathbf{0} \end{matrix} \right)\boldsymbol{\equiv}\mathbf{D}_{p}$$

Therefore,

$$\mathbf{S}_{t}^{L}\mathbf{=}\boldsymbol{\Gamma}^{T}\left( \mathbf{S}_{t}^{k}\boldsymbol{+}\lambda\mathbf{K}\boldsymbol{+}\alpha\mathbf{K}\mathbf{L}^{\phi}\mathbf{K} \right)\boldsymbol{\Gamma=}\boldsymbol{\Gamma}^{T}\left( \mathbf{T}_{1}^{-1} \right)^{T}\left( \mathbf{T}_{1}^{T}\left( \mathbf{S}_{t}^{k}\boldsymbol{+}\lambda\mathbf{K}\boldsymbol{+}\alpha\mathbf{K}\mathbf{L}^{\phi}\mathbf{K} \right)\mathbf{T}_{1} \right)\mathbf{T}_{1}^{-1}\boldsymbol{\Gamma=}{\tilde{\boldsymbol{\Gamma}}}^{T}\mathbf{D}_{r}\tilde{\boldsymbol{\Gamma}}$$

$$\mathbf{S}_{t}^{L}\mathbf{=}\boldsymbol{\Gamma}^{T}\mathbf{S}_{b}^{k}\boldsymbol{\Gamma=}\boldsymbol{\Gamma}^{T}\left( \mathbf{T}_{1}^{-1} \right)^{T}\left( \mathbf{T}_{1}^{T}\mathbf{S}_{b}^{k}\mathbf{T}_{1} \right)\mathbf{T}_{1}^{-1}\boldsymbol{\Gamma=}{\tilde{\boldsymbol{\Gamma}}}^{T}\mathbf{D}_{p}\tilde{\boldsymbol{\Gamma}}$$

Where $\tilde{\boldsymbol{\Gamma}}\boldsymbol{=}\mathbf{T}_{1}^{-1}\boldsymbol{\Gamma}$.

Let $\tilde{\boldsymbol{\Gamma}}\boldsymbol{=}\left( \begin{matrix} \boldsymbol{\Gamma}_{1} \\ \boldsymbol{\Gamma}_{2} \end{matrix} \right)$ be a partition of $\tilde{\boldsymbol{\Gamma}}$ so that $\boldsymbol{\Gamma}_{1}\in\mathbb{R}^{r\times s}$ and $\boldsymbol{\Gamma}_{2}\in\mathbb{R}^{(n-r)\times s}$. It follows that

$\mathbf{S}_{t}^{L}\mathbf{=}{\tilde{\boldsymbol{\Gamma}}}^{T}\mathbf{D}_{r}\tilde{\boldsymbol{\Gamma}}\boldsymbol{=}\boldsymbol{\Gamma}_{1}^{T}\boldsymbol{\Gamma}_{1}$, $\mathbf{S}_{b}^{L}\mathbf{=}{\tilde{\boldsymbol{\Gamma}}}^{T}\mathbf{D}_{p}\tilde{\boldsymbol{\Gamma}}\boldsymbol{=}\boldsymbol{\Gamma}_{1}^{T}\boldsymbol{\Sigma}_{F}\boldsymbol{\Sigma}_{F}^{T}\boldsymbol{\Gamma}_{1}$.

Hence，$F_{2}\mathbf{=}\mathrm{tr}\left( \left( \boldsymbol{\Gamma}^{T}\left( \mathbf{S}_{t}^{k}\boldsymbol{+}\lambda\mathbf{K}\boldsymbol{+}\alpha\mathbf{K}\mathbf{L}^{\phi}\mathbf{K} \right)\boldsymbol{\Gamma} \right)^{+}\boldsymbol{\Gamma}^{T}\mathbf{S}_{b}^{k}\boldsymbol{\Gamma} \right)=tr\left( {\boldsymbol{(\Gamma}_{1}^{T}\boldsymbol{\Gamma}_{1}\boldsymbol{)}}^{\boldsymbol{+}}\boldsymbol{(}\boldsymbol{\Gamma}_{1}^{T}\boldsymbol{\Sigma}_{F}\boldsymbol{\Sigma}_{F}^{T}\boldsymbol{\Gamma}_{1}\boldsymbol{)} \right)=tr(\left( {{\boldsymbol{\Gamma}_{1}\boldsymbol{\Gamma}}_{1}^{+}\boldsymbol{)}}^{T}\boldsymbol{\Sigma}_{F}\boldsymbol{\Sigma}_{F}^{T}\boldsymbol{(}{\boldsymbol{\Gamma}_{1}\boldsymbol{\Gamma}}_{1}^{+}\boldsymbol{)} \right)$

where the last equality follows from Lemma 1.

Recall that $\boldsymbol{\Sigma}_{F}\boldsymbol{\Sigma}_{F}^{T}\boldsymbol{=}\mathrm{diag}(\boldsymbol{\Sigma}_{p}^{2}\boldsymbol{,}\mathbf{0})$, by using a permutation matrix to order $\boldsymbol{\sum}_{F}\boldsymbol{=}\mathrm{diag}(\boldsymbol{\sum}_{p}\boldsymbol{,}\mathbf{0})$ (and likewise $\mathbf{U}_{F}$ ), we can assume that $\boldsymbol{\Sigma}_{F}\boldsymbol{\Sigma}_{F}^{T}\boldsymbol{=}\mathrm{diag}(\omega_{1}, \boldsymbol{\cdots,}\omega_{r})$ with $\omega_{1}\boldsymbol{\geq\cdots\geq}\omega_{p}\boldsymbol{>}0\boldsymbol{=}\omega_{p+1}=\boldsymbol{\cdots=}\omega_{r}$. Let $\boldsymbol{\Gamma}_{1}\boldsymbol{=}\breve{\mathbf{U}}\left( \begin{matrix} {\breve{\boldsymbol{\Sigma}}}_{\delta} & \mathbf{0} \\ \mathbf{0} & \mathbf{0} \end{matrix} \right){\breve{\mathbf{V}}}^{T}$ be the SVD of $\boldsymbol{\Gamma}_{1}$, where $\breve{\mathbf{U}}$ and $\breve{\mathbf{V}}$ are orthogonal, ${\breve{\boldsymbol{\Sigma}}}_{\delta}$ is diagonal, and $\delta=rank(\boldsymbol{\Gamma}_{1})$. Then $\boldsymbol{\Gamma}_{1}^{+}\boldsymbol{=}\breve{\mathbf{V}}\left( \begin{matrix} {\breve{\boldsymbol{\Sigma}}}_{\delta}^{-1} & \mathbf{0} \\ \mathbf{0} & \mathbf{0} \end{matrix} \right){\breve{\mathbf{U}}}^{T}$ and $\boldsymbol{\Gamma}_{1}\boldsymbol{\Gamma}_{1}^{+}\boldsymbol{=}\breve{\mathbf{U}}\left( \begin{matrix} \mathbf{I}_{\delta} & \mathbf{0} \\ \mathbf{0} & \mathbf{0} \end{matrix} \right){\breve{\mathbf{U}}}^{T}$. It follows that

$$F_{2}\mathbf{=}\mathrm{tr}\left( \left( {{\boldsymbol{\Gamma}_{1}\boldsymbol{\Gamma}}_{1}^{+}\boldsymbol{)}}^{T}\boldsymbol{\Sigma}_{F}\boldsymbol{\Sigma}_{F}^{T}\boldsymbol{(}{\boldsymbol{\Gamma}_{1}\boldsymbol{\Gamma}}_{1}^{+} \right) \right)=\mathrm{tr}\left( \breve{\mathbf{U}}\left( \begin{matrix} \mathbf{I}_{\delta} & \mathbf{0} \\ \mathbf{0} & \mathbf{0} \end{matrix} \right){\breve{\mathbf{U}}}^{T}\boldsymbol{\Sigma}_{F}\boldsymbol{\Sigma}_{F}^{T}\breve{\mathbf{U}}\left( \begin{matrix} \mathbf{I}_{\delta} & \mathbf{0} \\ \mathbf{0} & \mathbf{0} \end{matrix} \right){\breve{\mathbf{U}}}^{T} \right)$$

$$=\mathrm{tr}\left( \left( \begin{matrix} \mathbf{I}_{\delta} & \mathbf{0} \\ \mathbf{0} & \mathbf{0} \end{matrix} \right){\breve{\mathbf{U}}}^{T}\boldsymbol{\Sigma}_{F}\boldsymbol{\Sigma}_{F}^{T}\breve{\mathbf{U}}\left( \begin{matrix} \mathbf{I}_{\delta} & \mathbf{0} \\ \mathbf{0} & \mathbf{0} \end{matrix} \right) \right)=\mathrm{tr}\left( {\breve{\mathbf{U}}}_{\delta}^{T}\boldsymbol{\Sigma}_{F}\boldsymbol{\Sigma}_{F}^{T}{\breve{\mathbf{U}}}_{\delta} \right)\leq\omega_{1}\boldsymbol{+\cdots+}\omega_{p}$$

where ${\breve{\mathbf{U}}}_{\delta}$ is the matrix consisting of the first $\delta$ columns of $\breve{\mathbf{U}}$, and the last inequality follows from Lemma 2. By Lemma 2 again, the above inequality becomes equality, if ${\breve{\mathbf{U}}}_{\delta}\boldsymbol{=}\left( \begin{matrix} \boldsymbol{\Theta} \\ \mathbf{0} \end{matrix} \right)$, for any orthogonal $\boldsymbol{\Theta}\in\mathbb{R}^{p\times p}$, $\delta=p$, and *s*=*p*. Under this choice of ${\breve{\mathbf{U}}}_{\delta}$

$\boldsymbol{\Gamma}_{1}\boldsymbol{=}{{\breve{\mathbf{U}}}_{p}\breve{\boldsymbol{\Sigma}}}_{\delta}{\breve{\mathbf{V}}}^{T}\boldsymbol{=}\left( \begin{matrix} \boldsymbol{\Theta}{\breve{\boldsymbol{\Sigma}}}_{p}{\breve{\mathbf{V}}}^{T} \\ \mathbf{0} \end{matrix} \right)$.

We observe that the maximization of $F_{2}$ is independent of $\boldsymbol{\Gamma}_{2}$, and simply set it to zero. Therefore, the maximum of $F_{2}$ is attained when

$\tilde{\boldsymbol{\Gamma}}\boldsymbol{=}\left( \begin{matrix} \boldsymbol{\Gamma}_{1} \\ \boldsymbol{\Gamma}_{2} \end{matrix} \right)\boldsymbol{=}\left( \begin{matrix} \boldsymbol{\Theta}{\breve{\boldsymbol{\Sigma}}}_{p}{\breve{\mathbf{V}}}^{T} \\ \mathbf{0} \end{matrix} \right)$.

Note that the orthogonal matrices $\boldsymbol{\Theta}$ and $\breve{\mathbf{V}}$, and the diagonal matrix ${\breve{\boldsymbol{\Sigma}}}_{p}$ are arbitrary. Hence, $\boldsymbol{\Upsilon}=\boldsymbol{\Theta}{\breve{\boldsymbol{\Sigma}}}_{p}{\breve{\mathbf{V}}}^{T}$ is an arbitrary nonsingular matrix. It follows that $\boldsymbol{\Gamma=}\mathbf{T}_{1}\tilde{\boldsymbol{\Gamma}}\boldsymbol{=}\boldsymbol{T\Upsilon}$, for an arbitrary nonsingular $\boldsymbol{\Upsilon}$, maximizes $F_{2}$, where $\mathbf{T=}\mathbf{U}_{r}{\mathbf{P}_{r}\boldsymbol{\Lambda}}_{r}^{-1/2}\mathbf{U}_{p}$ consists of the first *p* columns of $\mathbf{T}_{1}$.

Since $\mathbf{T}^{T}\mathbf{KT}={(\boldsymbol{\Sigma}_{r}^{1/2}\mathbf{U}_{r}^{T}\mathbf{T})}^{T}(\boldsymbol{\Sigma}_{r}^{1/2}\mathbf{U}_{r}^{T}\mathbf{T})$, we have rank($\mathbf{T}^{T}\mathbf{KT}$)= rank($\boldsymbol{\Sigma}_{r}^{1/2}\mathbf{U}_{r}^{T}\mathbf{T}$)= rank($\mathbf{T}$)= *p*. This means that $\mathbf{T}^{T}\mathbf{KT}$ is positive definite (must be invertible). Let $\mathbf{T}^{T}\mathbf{KT=}\hat{\mathbf{U}}\hat{\boldsymbol{\Sigma}}{\hat{\mathbf{U}}}^{T}$ be the eigen-decomposition of $\mathbf{T}^{T}\mathbf{KT}$. It is easy to verify that $\hat{\boldsymbol{\sum}}$ is a diagonal matrix with positive entries on its diagonal.

Up to now, we have proved that for any nonsingular matrix $\boldsymbol{\Upsilon}$, $\boldsymbol{\Gamma}^{\boldsymbol{*}}\boldsymbol{=}\boldsymbol{T\Upsilon}$ maximizes the $F_{2}$ criterion. We have the freedom to choose $\boldsymbol{\Upsilon}$ to make the constraint in (16) satisfied. With $\boldsymbol{\Upsilon=}\hat{\mathbf{U}}{\hat{\boldsymbol{\Sigma}}}^{-1/2}$, ${\boldsymbol{\Upsilon}^{T}\mathbf{T}}^{T}\boldsymbol{KT\Upsilon=}{\hat{\boldsymbol{\Sigma}}}^{-1/2}{\hat{\mathbf{U}}}^{T}\hat{\mathbf{U}}\hat{\boldsymbol{\Sigma}}{\hat{\mathbf{U}}}^{T}\hat{\mathbf{U}}{\hat{\boldsymbol{\Sigma}}}^{-1/2}\boldsymbol{=}\mathbf{I}_{p}$. This completes the proof of the theorem.

**Appendix D: proof of Proposition 4**

*Proof:* Substituting $\boldsymbol{\Gamma}^{\boldsymbol{*}}\boldsymbol{=}\mathbf{T}\hat{\mathbf{U}}{\hat{\boldsymbol{\Sigma}}}^{-1/2}$ in Theorem 2 into (15) together with $\mathbf{T=}\mathbf{U}_{r}{\mathbf{P}_{r}\boldsymbol{\Lambda}}_{r}^{-1/2}\mathbf{U}_{p}$, we have

$$\mathbf{W}^{\phi}\boldsymbol{=}\left( {\hat{\boldsymbol{\Sigma}}}^{-1/2}{\hat{\mathbf{U}}}^{T}\mathbf{T}^{T}\left( \mathbf{K}^{2}\boldsymbol{+}\lambda\mathbf{K}\boldsymbol{+}\alpha\mathbf{K}\mathbf{L}^{\phi}\mathbf{K} \right)\mathbf{T}\hat{\mathbf{U}}{\hat{\boldsymbol{\Sigma}}}^{-1/2} \right)^{-1}{\boldsymbol{(}\boldsymbol{\Gamma}^{\boldsymbol{*}}\boldsymbol{)}}^{T}\mathbf{KY}$$

$$\boldsymbol{=}\left( {\hat{\boldsymbol{\Sigma}}}^{-1/2}{\hat{\mathbf{U}}}^{T}\mathbf{T}^{T}\mathbf{U}_{K}\left( \boldsymbol{\Sigma}_{K}^{2}\boldsymbol{+}\lambda\boldsymbol{\Sigma}_{K}\boldsymbol{+}\alpha\boldsymbol{\Sigma}_{K}\mathbf{U}_{K}^{T}\mathbf{L}^{\phi}\mathbf{U}_{K}\boldsymbol{\Sigma}_{K} \right)\mathbf{U}_{K}^{T}\mathbf{T}\hat{\mathbf{U}}{\hat{\boldsymbol{\Sigma}}}^{-1/2} \right)^{-1}{\boldsymbol{(}\boldsymbol{\Gamma}^{\boldsymbol{*}}\boldsymbol{)}}^{T}\mathbf{KY}$$

$$\boldsymbol{=}\left( {\hat{\boldsymbol{\Sigma}}}^{-1/2}{\hat{\mathbf{U}}}^{T}\mathbf{T}^{T}\mathbf{U}_{K}\left( \left( \begin{matrix} \boldsymbol{\Sigma}_{r}^{2} & \mathbf{0} \\ \mathbf{0} & \mathbf{0} \end{matrix} \right)\boldsymbol{+}\left( \begin{matrix} \lambda\boldsymbol{\Sigma}_{r} & \mathbf{0} \\ \mathbf{0} & \mathbf{0} \end{matrix} \right)\boldsymbol{+}\left( \begin{matrix} \alpha\boldsymbol{\Sigma}_{r}\mathbf{U}_{r}^{T}\mathbf{L}^{\phi}\mathbf{U}_{r}\boldsymbol{\Sigma}_{r} & \mathbf{0} \\ \mathbf{0} & \mathbf{0} \end{matrix} \right) \right)\mathbf{U}_{K}^{T}\mathbf{T}\hat{\mathbf{U}}{\hat{\boldsymbol{\Sigma}}}^{-1/2} \right)^{-1}{\boldsymbol{(}\boldsymbol{\Gamma}^{\boldsymbol{*}}\boldsymbol{)}}^{T}\mathbf{KY}$$

$$\boldsymbol{=}\left( {\hat{\boldsymbol{\Sigma}}}^{-1/2}{\hat{\mathbf{U}}}^{T}\mathbf{T}^{T}\boldsymbol{[}\mathbf{U}_{\boldsymbol{r}}\boldsymbol{,}\mathbf{U}_{r}^{\perp}\boldsymbol{]}\left( \begin{matrix} \mathbf{U}_{r}{(\boldsymbol{\Sigma}_{r}^{2}+\lambda\boldsymbol{\Sigma}_{r}+\alpha\boldsymbol{\Sigma}_{r}\mathbf{U}_{r}^{T}\mathbf{L}^{\phi}\mathbf{U}_{r}\boldsymbol{\Sigma}_{r}\boldsymbol{)}}^{-1} \mathbf{U}_{r}^{T} & \mathbf{0} \\ \mathbf{0} & \mathbf{0} \end{matrix} \right){\boldsymbol{[}\mathbf{U}_{\boldsymbol{r}}\boldsymbol{,}\mathbf{U}_{r}^{\perp}\boldsymbol{]}}^{T}\mathbf{T}\hat{\mathbf{U}}{\hat{\boldsymbol{\Sigma}}}^{-1/2} \right)^{-1}{\boldsymbol{(}\boldsymbol{\Gamma}^{\boldsymbol{*}}\boldsymbol{)}}^{T}\mathbf{KY}$$

$$\boldsymbol{=}\left( {\hat{\boldsymbol{\Sigma}}}^{-1/2}{\hat{\mathbf{U}}}^{T}\mathbf{T}^{T}\mathbf{U}_{\boldsymbol{r}}(\boldsymbol{\Sigma}_{r}^{2}+\lambda\boldsymbol{\Sigma}_{r}+\alpha\boldsymbol{\Sigma}_{r}\mathbf{U}_{r}^{T}\mathbf{L}^{\phi}\mathbf{U}_{r}\boldsymbol{\Sigma}_{r})\mathbf{U}_{r}^{T}\mathbf{T}\hat{\mathbf{U}}{\hat{\boldsymbol{\Sigma}}}^{-1/2} \right)^{-1}{\boldsymbol{(}\boldsymbol{\Gamma}^{\boldsymbol{*}}\boldsymbol{)}}^{T}\mathbf{KY}$$

$$\boldsymbol{=}\left( {\hat{\boldsymbol{\Sigma}}}^{-1/2}{\hat{\mathbf{U}}}^{T}\mathbf{T}^{T}\mathbf{U}_{\boldsymbol{r}}{{\tilde{\boldsymbol{\Sigma}}}_{r}\mathbf{U}}_{r}^{T}\mathbf{T}\hat{\mathbf{U}}{\hat{\boldsymbol{\Sigma}}}^{-1/2} \right)^{-1}{\boldsymbol{(}\boldsymbol{\Gamma}^{\boldsymbol{*}}\boldsymbol{)}}^{T}\mathbf{KY}$$

$$\boldsymbol{=}\left( {\hat{\boldsymbol{\Sigma}}}^{-1/2}{\hat{\mathbf{U}}}^{T}\mathbf{T}^{T}\mathbf{U}_{\boldsymbol{r}}{{\mathbf{P}_{r}\boldsymbol{\Lambda}_{r}\mathbf{P}}_{r}^{T}\mathbf{U}}_{r}^{T}\mathbf{T}\hat{\mathbf{U}}{\hat{\boldsymbol{\Sigma}}}^{-1/2} \right)^{-1}{\boldsymbol{(}\boldsymbol{\Gamma}^{\boldsymbol{*}}\boldsymbol{)}}^{T}\mathbf{KY}$$

$$\boldsymbol{=}\left( {\hat{\boldsymbol{\Sigma}}}^{-1/2}{\hat{\mathbf{U}}}^{T}\mathbf{U}_{p}^{T}{\boldsymbol{\Lambda}_{r}^{-1/2}\mathbf{P}_{r}^{T}\mathbf{U}_{r}^{T}\mathbf{U}}_{\boldsymbol{r}}{{\mathbf{P}_{r}\boldsymbol{\Lambda}_{r}\mathbf{P}}_{r}^{T}\mathbf{U}}_{r}^{T}\mathbf{U}_{\boldsymbol{r}}\mathbf{P}_{r} \boldsymbol{\Lambda}_{r}^{-1/2}\mathbf{U}_{\boldsymbol{p}}\hat{\mathbf{U}}{\hat{\boldsymbol{\Sigma}}}^{-1/2} \right)^{-1}{\boldsymbol{(}\boldsymbol{\Gamma}^{\boldsymbol{*}}\boldsymbol{)}}^{T}\mathbf{KY}$$

$$\mathbf{=}\hat{\boldsymbol{\Sigma}}\mathbf{(}{\mathbf{T}\hat{\mathbf{U}}{\hat{\boldsymbol{\Sigma}}}^{-1/2}\mathbf{)}}^{T}\mathbf{KY=}{\hat{\boldsymbol{\Sigma}}}^{1/2}{{\hat{\mathbf{U}}}^{T}\mathbf{T}}^{T}\mathbf{KY}$$
